# Dynamic phosphorylation of RelA on Ser42 and Ser45 in response to TNFα stimulation regulates DNA binding and transcription

**DOI:** 10.1098/rsob.160055

**Published:** 2016-07-27

**Authors:** Francesco Lanucara, Connie Lam, Jelena Mann, Tom P. Monie, Stefano A. P. Colombo, Stephen W. Holman, James Boyd, Manohar C. Dange, Derek A. Mann, Michael R. H. White, Claire E. Eyers

**Affiliations:** 1 Centre for Proteome Research, Department of Biochemistry, Institute of Integrative Biology, University of Liverpool, Crown Street, Liverpool L69 7ZB, UK; 2 Systems Microscopy Centre, Faculty of Life Sciences, University of Manchester, Manchester M13 9PT, UK; 3 Fibrosis Laboratory, Liver Group, Institute of Cellular Medicine, Newcastle University, Newcastle upon Tyne NE2 4HH, UK; 4 MRC Human Nutrition Research, University of Cambridge, Cambridge CB2 1GA, UK

**Keywords:** NF-κB, phosphorylation, RelA, proteomics, quantification, transcription

## Abstract

The NF-κB signalling module controls transcription through a network of protein kinases such as the IKKs, as well as inhibitory proteins (IκBs) and transcription factors including RelA/p65. Phosphorylation of the NF-κB subunits is critical for dictating system dynamics. Using both non-targeted discovery and quantitative selected reaction monitoring-targeted proteomics, we show that the cytokine TNFα induces dynamic multisite phosphorylation of RelA at a number of previously unidentified residues. Putative roles for many of these phosphorylation sites on RelA were predicted by modelling of various crystal structures. Stoichiometry of phosphorylation determination of Ser45 and Ser42 revealed preferential early phosphorylation of Ser45 in response to TNFα. Quantitative analyses subsequently confirmed differential roles for pSer42 and pSer45 in promoter-specific DNA binding and a role for both of these phosphosites in regulating transcription from the IL-6 promoter. These temporal dynamics suggest that RelA-mediated transcription is likely to be controlled by functionally distinct NF-κB proteoforms carrying different combinations of modifications, rather than a simple ‘one modification, one effect’ system.

## 1. Introduction

The transcription factor RelA (also known as p65) is a key component of NF-κB signalling. Upon dimerization, RelA regulates expression of a myriad of genes underpinning diverse cellular processes, including inflammation, cell growth and transformation. Impairment or dysregulation of the system has been linked to many severe pathological states, including chronic inflammatory diseases [1], autoimmune disorders [2] and cancer [3]. Like most signalling pathways, the dynamics of NF-κB is precisely controlled by post-translational modifications (PTMs), with phosphorylation playing a central rate-limiting role in transcription [4–6]. Modification of approximately 25 different RelA residues has been reported to date, although many have been annotated solely following high-throughput mass spectrometric analysis [7] (www.phosphosite.org [8]). Distinct phosphorylation events are known to modulate RelA stability, nuclear localization and/or transcriptional activity by regulating binding to DNA and/or proteins [5]. It is now appreciated that the prevalence of different NF-κB modifications, that constitute the so-called NF-κB code [9], are likely to be dependent on both the context and activating stimulus, and the specific cell type.

Elucidation of the mechanistic control of NF-κB signalling outputs in response to specific extracellular signals requires an understanding of the roles and interplay of a dense network of coexisting RelA modifications. Most studies examining covalent RelA modification evaluate select sites of interest, often relying on mutagenesis or site-specific antibodies to study individual sites. While these strategies have generated much fundamental information, they fail to examine the composite, biologically relevant picture of the dynamics and roles of the different post-translationally modified forms (proteoforms) of the NF-κB complex. An in-depth investigation of RelA phosphorylation under defined cellular conditions is still lacking, yet such studies are essential to understand the combinatorial role of different modifications. Crucially, this lack of information significantly impairs our ability to model the NF-κB system at levels approaching the natural complexity found *in vivo* [10,11].

Mass spectrometry (MS) is the method of choice for PTM characterization owing to its sensitivity and versatility, offering unique advantages over other approaches. MS permits the identification and quantification of modification sites even under extremely challenging conditions, for example when they occur with low stoichiometry or are located on low abundance proteins such as transcription factors. However, the substoichiometric nature of most PTM events reduces the likelihood of their identification in a typical shotgun data-dependent acquisition (DDA), where only the most abundant peptide ions will yield sequence information. While these types of experiments are extremely useful for discovery purposes, more targeted approaches, often based on selected reaction monitoring (SRM), improve detection and are optimal for the quantification of (modified) peptides [12–14].

Cellular exposure to cytokines such as tumour necrosis factor alpha (TNFα) induces IKK-mediated phosphorylation of IκB, targeting it for degradation via the ubiquitin–proteosome pathway (reviewed in [15]). Consequently, activated NF-κB is no longer sequestered in the cytoplasm and can translocate to the nucleus, where it regulates target gene transcription. Prolonged stimulation with TNFα promotes cell type-dependent sustained nuclear–cytoplasmic oscillations of RelA with a typical period of approximately 100 min, regulated by negative feedback loops involving the continual degradation and re-synthesis of IκB via the ‘canonical’ pathway [16–19]. Although RelA is known to be phosphorylated on a number of residues following cytokine stimulation, the lack of consistency in experimental design makes understanding phosphosite dynamics, their coordinated regulation and physiological functions nigh-on impossible. Here, we exploit a combination of shotgun and targeted MS strategies to define the temporal dynamics of endogenous RelA phosphorylation in SK-N-AS neuroblastoma cells in response to TNFα exposure, identifying seven novel phosphorylation sites on RelA (Ser42, Ser131, Thr136, Ser238, Ser261, Ser269 and Ser472). Additionally, we characterize a biologically relevant phosphosite (Ser45) only previously annotated in high-throughput proteomic analyses. Structural interrogation and cell-based analyses enable us to confirm that two of these phosphorylation sites, Ser42 and Ser45, regulate DNA binding and transcription. Furthermore, our data provide the first quantitative temporal fingerprint of RelA phosphorylation dynamics, information that will be vital to understand, model and ultimately selectively perturb the NF-κB signalling module.

## 2. Results

### 2.1. Endogenous RelA is dynamically phosphorylated following cellular stimulation with TNFα

**Figure 1.**
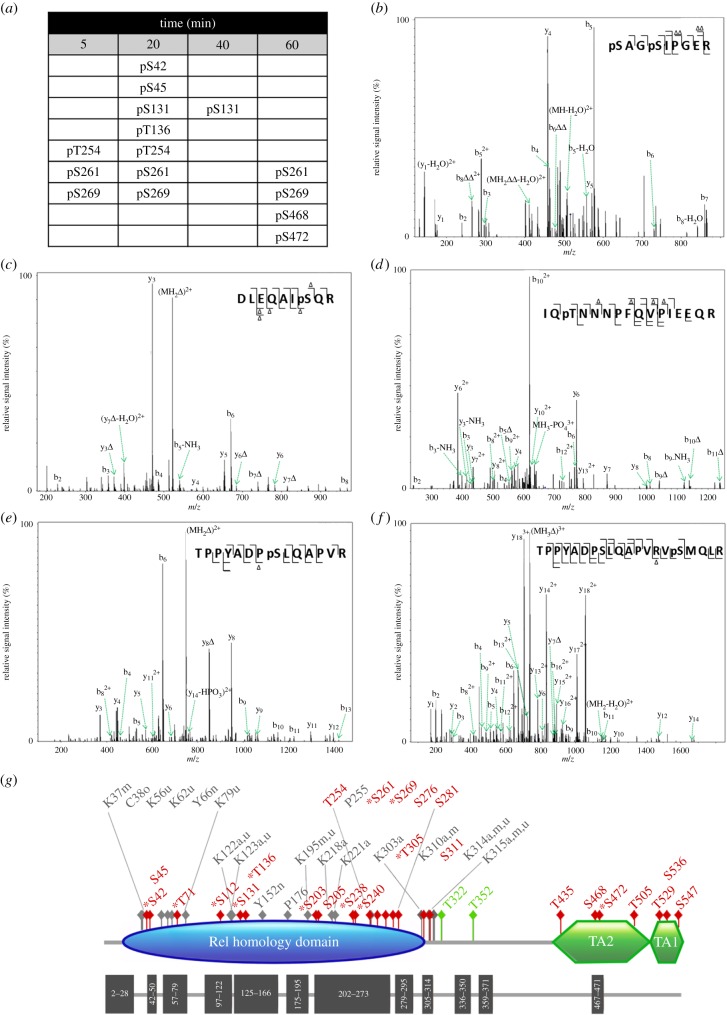
TNFα induces dynamic multi-site phosphorylation of endogenous RelA. (*a*) Phosphorylation sites identified at 5, 20, 40, 60 min post-stimulation of SK-N-AS cells with the cytokine TNFα are detailed. No phosphorylation sites were observed in the absence of stimulation. CID product ion spectra of a (*b*) doubly charged ion at *m/z* 437.7, indicating phosphorylation of Ser42 and Ser45; (*c*) doubly charged ion at *m/z* 570.1, indicating phosphorylation of Ser131; (*d*) triply charged ion at *m/z* 669.7, indicating phosphorylation of Ser136; (*e*) doubly charged ion *m/z* 796.2*,* indicating phosphorylation of Ser261; (*f*) triply charged ion at *m/z* 769.2*,* indicating phosphorylation of Ser269. (*g*) Schematic of RelA detailing known and novel (*) sites of modification. Phosphorylation sites are in red, glycosylation sites in green; a, acetylation; m, methylation; n, nitrosylation; o, oxidation; u, ubiquitination; TA, transactivation domain. Dark grey blocks represent those regions in the primary sequence identified by shotgun LC–MS/MS analysis following proteolytic cleavage with different enzymes.

Our initial objective was to dissect dynamic (stimulation-induced) regulation of RelA by PTMs. We first set out to characterize the sites of modification on endogenous RelA following exposure of human cells to the pro-inflammatory cytokine TNFα. Preliminary analyses of a tryptic digest of SK-N-AS cell extracts fractionated either by strong anion exchange (36 fractions), high-pH reversed-phase (40 fractions) or GeLC (40 slices from SDS–PAGE) [20] prior to LC–MS/MS failed to identify RelA-derived tryptic peptides, indicating that the endogenous transcription factor was expressed at levels below the detection limit of the nano-ESI-Orbitrap Velos system used, validated here to be in the region of 120 000 protein copies (approx. 200 zeptomoles) per cell [21], owing to limitations in the number of cells that can be analysed in a single run. All subsequent investigations thus relied on antibody-based enrichment of endogenous RelA. LC–MS/MS analysis of tryptic peptides derived from immunoprecipitated (IP'ed) RelA yielded 52% sequence coverage, with peptides representing most of the protein N-terminus (amino acids 42–314; figure 1), but lacking coverage over the C-terminus. Of note for ‘bottom-up’ proteomics analysis and PTM discovery, much of the RelA C-terminal region, which contains the transactivation domains (TAs), lacks suitable Lys/Arg trypsin cleavage sites. Indeed, *in silico* tryptic digestion of RelA predicts that approximately 40% of theoretical peptides will not be observed, primarily because the peptide fragments are too large to elute from a standard C_18_ reverse-phase column. Moreover, many of the potential tryptic cleavage sites are either clustered in basic (e.g. KKK or KxKxK) or acidic (D/E at positions P2, P1′ and P2′) motifs, which significantly increase the likelihood of incomplete proteolysis [22–24]. Incomplete trypsin digestion of NF-κB proteins is further complicated by extensive PTM, including Ser/Thr phosphorylation and Lys acetylation, both of which decrease proteolytic efficiency [25]. To overcome this problem, alternate site-specific proteases (chymotrypsin, elastase, GluC) were employed, and these marginally increased protein sequence coverage. When combined with tryptic digest information, this led to the identification of nine sites of RelA phosphorylation after TiO_2_ phosphopeptide-enrichment, including six novel sites: pSer42, pSer131, pThr136, pSer261, pSer269 and pSer472 (figure 1 and electronic supplementary material, table S1 and figure S1). In addition, the phosphorylation of Ser45, a site previously reported in a single high-throughput MS-based screen [26], was confirmed, and two known sites of phosphorylation, pThr254 [27] and pSer468 [28–30], were identified (electronic supplementary material, figure S1).

Interestingly, this extensive analysis still did not account for several TNFα-induced phosphorylation sites on RelA originally identified using non-MS-based approaches, including pSer536 [29,31,32], most likely owing to an inability to generate suitable peptides for analysis under these conditions. Although we interrogated the LC–MS data for other types of PTM, including Cys oxidation, sulfhydration, nitrosylation, acetylation and methylation of Lys, Tyr nitrosylation and ubiquitinylation [33–35], only a single acetylation site at Lys303 was observed.

### 2.2. p50-dependent hyperphosphorylation of RelA is abrogated by IκBα

**Figure 2.**
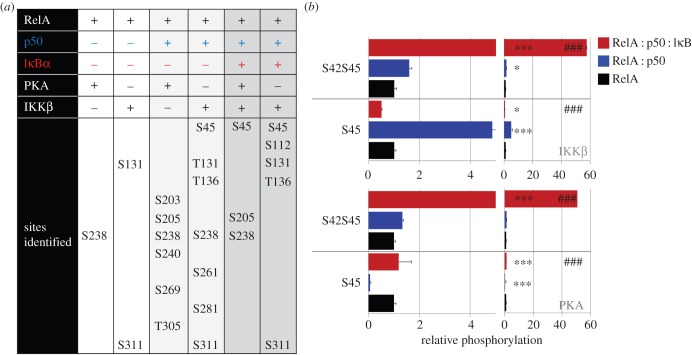
*In vitro* site-specific RelA phosphorylation is significantly increased in the presence of IκBα and/or p50. The sites identified following *in vitro* phosphorylation of RelA with PKA or IKKβ, either alone or in the presence of stoichiometric amounts of p50, or p50 and IκBα are detailed. (*a*) Qualitative phosphosite identification following discovery LC–MS/MS. (*b*) Selected reaction monitoring (SRM) determined relative change in phosphopeptide level compared with RelA alone. Statistical significance was assessed using a one-way ANOVA and a post hoc Tukey test; *p < 0.05, ***p < 0.001 with respect to RelA alone for each site and kinase; ###*p* < 0.001 with respect to RelA : p50 for each site and kinase. N.B. Relative phosphorylation cannot be directly compared between phosphopeptides and thus phosphorylation sites owing to differences in peptide ionization efficiency.

To help understand the regulation of RelA phosphorylation using a biochemical approach and purified components, LC–MS/MS was employed to map phosphorylation sites on RelA (12–317) induced by the known NF-κB kinases IKKβ and the catalytic subunit of protein kinase A (PKA; figure 2) [30,36]. In an attempt to mimic the RelA supramolecular assemblies found in cells, phosphorylation was also monitored after inclusion of stoichiometric amounts of the IκBα and/or p50 partner proteins (electronic supplementary material, figures S1–S3). In addition to the discovery studies used to pinpoint the residues phosphorylated under each of these conditions, quantitative SRM assays were developed to assess stoichiometry of phosphorylation of each of the sites, and therefore determine the relative change in phosphorylation upon inclusion of p50 and IκBα (figure 2 and electronic supplementary material, table S2 and figure S3). SRM assays that target specific peptide ions overcome the stochastic nature of data-dependent MS-based proteomics, where lack of observation of a (phospho)peptide is not necessarily indicative of its absence.

IKKβ induced RelA phosphorylation on both the newly identified Ser131 and at Ser311 (figure 2 and electronic supplementary material, figure S3), a site previously reported to be PKC-dependent [37,38]. Inclusion of p50, a key RelA heterodimerization partner, increased levels of both pSer131 and pSer311 by threefold and 1.6-fold, respectively. The increase in phosphorylation at both sites was markedly reduced (approx. 7- and 12.5-fold for Ser131 and Ser311, respectively) upon inclusion of IκBα with the RelA : p50 complex. Likewise, the PKA and/or IKKβ-mediated phosphorylation of Ser238 was abrogated in the presence of p50 and IκBα.

When evaluating relative phosphorylation at Ser45, it was important to consider total amounts of both the singly phosphorylated pSer45-containing, as well as the doubly phosphorylated Ser42/Ser45 peptides. IKKβ phosphorylation of the singly phosphorylated Ser45 RelA peptide increased significantly in the presence of p50, but there was a notable reduction in the levels of this phosphopeptide upon addition of IκBα (figure 2). When considering these data alongside the marked increase in the levels of the doubly phosphorylated Ser42/Ser45-containing peptide, it is apparent that phosphorylation of both Ser42 and Ser45 is enhanced upon formation of the tertiary complex, equivalent to approximately 60-fold over RelA alone. Like IKKβ, PKA phosphorylation of Ser42/Ser45 is significantly elevated in the ternary complex of RelA : p50 : IκBα. However, unlike IKKβ, PKA favours phosphorylation of both Ser42 and Ser45 on RelA for the RelA : p50 heterodimer. pSer45 is thus maximal in the presence of p50 and IκBα, irrespective of the kinase used.

The levels of pSer261, pSer281 and pThr305, in addition to pSer131 and pSer311, increased significantly in the presence of p50 following IKKβ incubation, but were reduced (or absent) when co-incubated with IκBα. p50 thus promotes hyperphosphorylation of RelA by IKKβ, and to a lesser extent PKA, and this is abrogated upon addition of the inhibitor IκBα protein. Although only three (Ser205, Ser281 and Ser311) of the 13 phosphosites identified in our *in vitro* studies have previously been described [36,37,39–41], four new *in cellulo* sites (Ser131, Thr136, Ser261, Ser269) were validated in these biochemical assays (electronic supplementary material, table S1).

### 2.3. Structural modelling predicts putative roles for RelA phosphorylation sites

To assess functional conservation of RelA phosphosites in vertebrates, a sequence alignment was performed. Of the 17 phosphorylation sites identified (combined *in vitro* and *in vivo*), nine residues (five of which have not previously been described (in italics)) are completely conserved across all species (*Ser42,* Ser45*, Ser112, Thr136, Ser203*, Ser205, Thr254, Ser281, *Thr305*), as is the previously reported Ser276 (electronic supplementary material, figure S3). *Ser238, Ser240* and *Ser472* (and previously identified sites at Ser311, Ser468, Thr505, Ser529, Ser536 and Ser547) are highly conserved in most mammals, but exhibit reduced conservation in chicken, frog and zebrafish. It is interesting to note that sequence conservation is considerably higher across the N-terminal RHD, which regulates binding to proteins such as the NF-κB subunits, when compared with the C-terminal TA domains.

**Figure 3.**
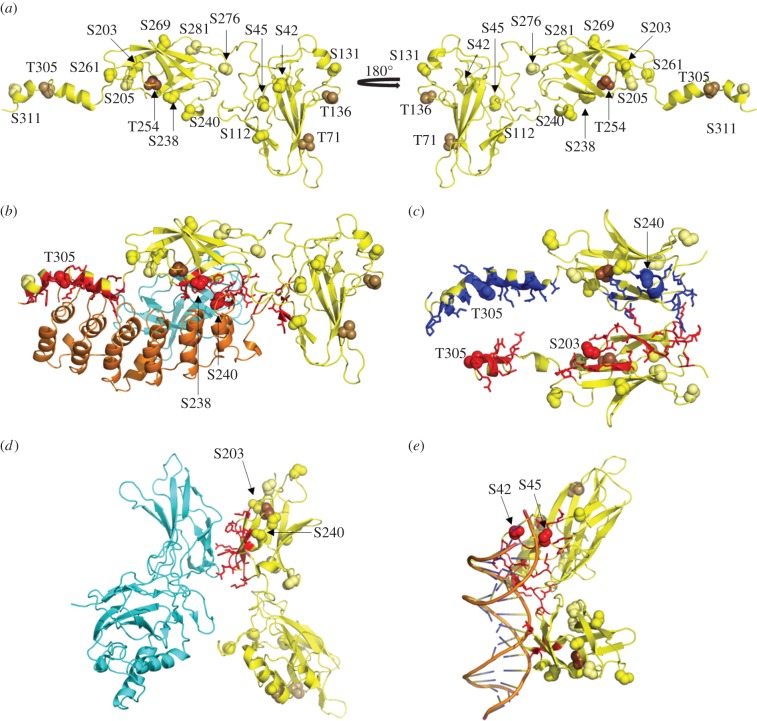
Structural representations of RelA. (*a*–*e*) Cartoon of RelA and interfaces with specific interaction partners. Phosphorylated residues are depicted as spheres and images are coloured as follows: RelA, yellow; p50, cyan; IκBα, orange; interface residues on RelA, red or blue; novel serine phosphorylation sites, yellow; known serine phosphorylation sites, pale yellow; novel threonine phosphorylation sites, light brown; known threonine phosphorylation sites, brown. (*a*) Location of phosphorylation sites on RelA (from PDB ID: 1IKN [43]). (*b*) RelA interface with IκBα (PDB ID: 1NFI [42]). (*c*) Interface residues on the RelA homodimer with IκBβ (PDB ID:1K3Z [44]—IκBβ removed for clarity). (*d*) RelA : p50 heterodimer (PDB ID: 1LEI [45]—DNA strand removed for clarity) (*e*) RelA contacts with the κB DNA sequence (PDB ID: 1LEI—p50 removed for clarity).

Co-crystal structures of RelA in complex with p50, IκB and/or DNA, including PDB IDs: 1NFI (RelA : p50 : IκBα) [42], 1IKN (RelA : p50 : IκBα) [43], 1K3Z (RelA : IκBβ) [44] and 1LEI (RelA : p50 : DNA) [45] were employed to evaluate potential roles of RelA phosphorylation on intermolecular complex assembly. Notably Ser240, which lies at the interface with IκB (α/β), acts to stabilize the interaction of the complex by formation of hydrogen bonds with either Arg260 (1IKN) or Leu256 (1NFI) of IκBα (figure 3*a*,*b*). Phosphorylation at Ser240 is predicted to destabilize the interaction of RelA with IκBα by removing these interactions. Close proximity of Ser238 to residues regulating the interaction with IκBα through either hydrogen bonds (Ser240, Gln241, Asp243) or a salt bridge (Asp243) also supports a role for pSer238 in regulating the interaction of RelA with IκBα. Furthermore, this modelling supports our *in vitro* kinase assay data, where statistically lower levels (0.7-fold) of IKKβ-induced pSer238 in the presence of IκBα (figure 2) were measured. Likewise, phosphorylation of RelA at Ser261, which also lies near the interface with IκBα, was 2.1-fold higher in the absence of IκBα. Structural comparison of both a RelA homodimer complexed with IκBβ (1K3Z [44] and figure 3*c*) and a RelA : p50 heterodimer with IκBα suggests that pSer203 and pThr305 also are likely to regulate the RelA : IκB interaction. The interface between RelA, p50 and p105 lies ±5 residues either side of Ser205 (figure 3*d*), indicating a role for pSer205 (and potentially pSer203) in regulating heterodimer formation. Of particular interest were Ser42 and Ser45, given that they appear to be directly involved in the interaction of NF-κB with the κB DNA sequence (figure 3*e*). We thus predicted a critical role for these sites in regulating the DNA binding propensity of RelA.

### 2.4. Quantitative selected reaction monitoring-based temporal profiling of Ser42 and Ser45 phosphorylation in response to TNFα

The DNA binding and transcriptional activity of NF-κB subunits is known to be dynamically regulated by reversible covalent modifications [27–29,31,46–54]. Our observation that, first, phosphorylation at Ser42 and Ser45 appears to be temporally regulated in response to TNFα (figure 1), second that both Ser42 and Ser45 are evolutionarily conserved (electronic supplementary material, figure S3) and third that phosphorylation of one or both of these sites may structurally regulate DNA binding (figure 3*e*) meant that defining and quantifying TNFα-induced temporal dynamics of phosphorylation of these two residues was a priority. SRM, exploiting isotope-labelled peptides as internal reference standards, offers significant improvements in detection limits over the discovery-type analyses used for early RelA phosphosite mapping [14] and is ideally suited to quantitative temporal profiling. Quantification by SRM is the ‘gold-standard’ in protein quantification and is significantly more robust than other commonly exploited techniques such as western blotting [55].

**Figure 4.**
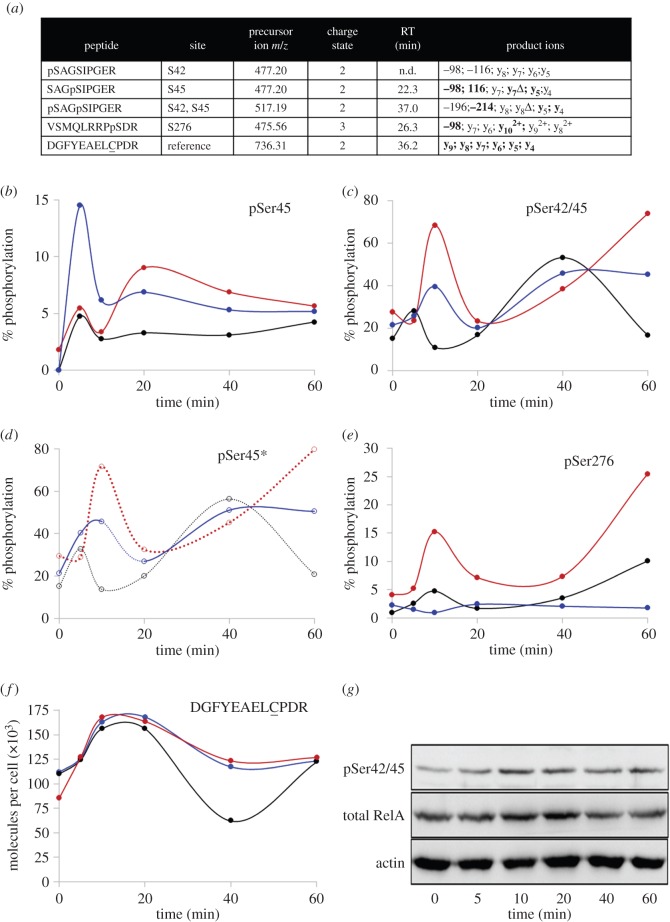
Selected-reaction monitoring (SRM) of RelA phosphorylation in response to treatment of SK-N-AS cells with the cytokine TNFα. (*a*) List of targeted (phospho)peptides included in the SRM analysis. Sequences of peptides, including phosphorylation sites (p), are listed along with their respective precursor ion *m/z* values, precursor ion charge state, retention time (RT) and product ions. C represents carbamidomethylated cysteine; −98 indicates loss of H_3_PO_4_ from the precursor ion; −116 indicates loss of (H_3_PO_4_ + H_2_O) from precursor ion; n.d. not detected; consistently observed product ions are highlighted in bold. (Phospho)peptides containing modified residues pSer45 (*b*), pSer42/45 (*c*), pSer45* (*d*), pSer276 (*e*) and the RelA control peptide DGFYEAELCPDR, where C denotes carbamidomethylation of Cys (*f*) were quantified by scheduled LC–SRM analysis following stimulation of SK-N-AS cells with TNFα; stimulation and analysis was repeated in triplicate for three different biological replicates represented in black, blue and red. Isotope-labelled internal reference (phospho)peptides were included, permitting absolute peptide quantification and phosphorylation site stoichiometry determination. pSer45* is the calculated stoichiometry of the Ser45 phosphorylation site obtained by summating percentage phosphorylation of the pSer45 and pSer42/45 phosphopeptides. Immunoblot analysis of pSer42/45 (*g*) is shown as a function of time following TNFα stimulation with reference to total RelA levels and actin control.

We designed SRM assays for singly (pSer42 or pSer45) and doubly phosphorylated (pSer42 and pSer45) forms of the tryptic peptide (p)SAG(p)SIPGER, as well as the sequence surrounding pSer276 (VSMQLRRPpSDR). SRM transitions were also designed for a non-modified reference peptide (DGFYEAELCPDR), whose quantitative profiling was used to normalize for the total amount of IP'ed endogenous RelA in the assays (figure 4*a* and electronic supplementary material, figures S4 and S5). Phosphopeptide (and thus phosphosite) quantification as a function of time following cell stimulation was performed by monitoring the SRM signals (peak areas) of the three phosphopeptides relative to the non-modified RelA tryptic peptide. In order to define the absolute amounts of each of the phosphopeptides, and thus the stoichiometry of phosphorylation at each of the sites, known amounts of identical [^13^C_6_] Arg peptides were included as internal quantification standards.

Using this approach, quantitative changes in site-specific RelA phosphorylation stoichiometry *in cellulo* were determined for the first time. As well as confirming cellular phosphorylation of Ser238, we evaluated time-dependent pSer45, pSer42pSer45 and pSer276 modification after TNFα exposure, employing three independent biological replicates for analysis (figure 4*b*–*e*). A series of co-eluting transitions specific for the peptide phosphorylated at Ser45 (including y_7_–98) were recorded, permitting discrimination between pSer42 and pSer45 positional isomers and quantification of pSer45.

SRM-based quantification of endogenous RelA phosphorylation reveals rapid biphasic regulation of pSer42/45 in response to TNFα stimulation (figure 4). Similar phosphorylation dynamics were also observed after immunoblotting with a phospho-specific antibody raised against the dual Ser42/45 phosphopeptide (figure 4*g*).

In order to define the stoichiometry and dynamics of phosphorylation at Ser45, it was important to co-evaluate the singly and doubly phosphorylated peptides covering pSer45 and pSer42/45 (figure 4*b*–*d*). Total pSer45 levels (pSer45*) were calculated by summation of the percentage phosphorylation of the two pSer45-containing phosphopeptides for each biological replicate (figure 4*d*). Although the singly phosphorylated pSer45-containing peptide peaked 5 min after TNFα stimulation, subsequently rising again at later time points (figure 4*b*), there was a consistent delay in pSer42/45 phosphopeptide dynamics, as determined by SRM (and validated by western blotting), indicating that Ser45 phosphorylation occurs first. Indeed, Ser42 was never observed in its phosphorylated form in the absence of pSer45 under these conditions. Phosphorylation of Ser45 (pSer45*, figure 4*d*) clearly demonstrates cyclical dynamics, with phosphorylation peaking rapidly after stimulation (approx. 5–10 min) before decreasing to basal levels (20 min) and then rising to maximal values after 40–60 min. Interestingly, this is similar in timing to the dynamics observed for the pSer276-containing peptide (figure 4*e*), a phosphorylation site previously demonstrated to be essential for transcription from a subset of NF-κB-dependent genes, exemplified by IL-8 transcription [36,39,56,57].

The biological variance in phosphosite dynamics identified between our biological replicates can be explained, in large part, by the discrete rather than by continuous time-points employed in our assays: note the much faster initial rate of pSer42/45 for a single bioreplicate (figure 4*c*, black) which consequently shifts the cycling phosphorylation levels earlier. Perhaps unsurprisingly, the precise stoichiometry of phosphorylation at each time point is not identical for the individual bioreplicate samples, suggesting that it is the variation or fold-change in levels of a given phosphorylation site that mediates stimulation-induced responses rather than the absolute stoichiometry of modification.

### 2.5. Rapid RelA phosphorylation at Ser42/45 regulates DNA binding and transcription

**Figure 5.**
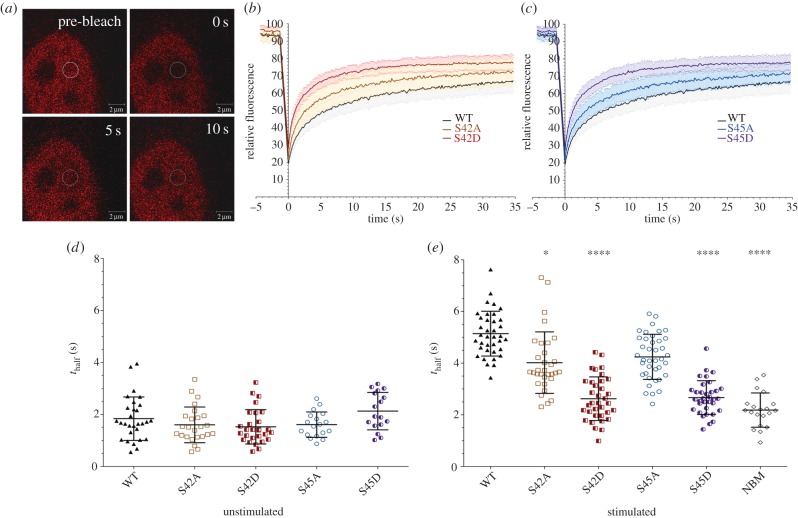
Nuclear movement of S42/S45 phosphomimetic versions of RelA–DsRed is increased. Fluorescent recovery after photobleaching (FRAP) was used to assess nuclear movement of RelA–DsRed species (wild-type (WT), S42A, S42D, S45A, S45D) in SK-N-AS cells. Cells were unstimulated (control) or stimulated with TNFα (10 ng µl^−1^) for 30 min prior to bleaching of a 3.14 µm^2^ (2 µm diameter) region of interest (ROI). (*a*) Single captures of a representative SK-N-AS cell nucleus with WT RelA–DsRed: prior to bleaching and at 0, 5 and 10 s post-bleaching. Images show recovery of fluorescence into the ROI following bleaching. (*b*,*c*) Mean fluorescent recovery curves of WT, S42A/S42D (*b*) and S45A/S45D (*c*) RelA–DsRed species. Error bars represent standard deviation (*σ*). All curves are significantly different from one another (*p* < 0.0001). (*d*,*e*) Scatter dot plots of the half time to recovery, *t*_half_ (s), of individual RelA–dsRed species in the cytoplasm of unstimulated cells (*d*) or in the nucleus following TNFα treatment (*e*). NBM refers to the non-binding mutant (Y36A/E39D) of RelA. The Kruskal–Wallis test was used with a Dunn's multiple comparison test to determine statistical difference: *p < 0.1; ****p < 0.0001.

**Figure 6.**
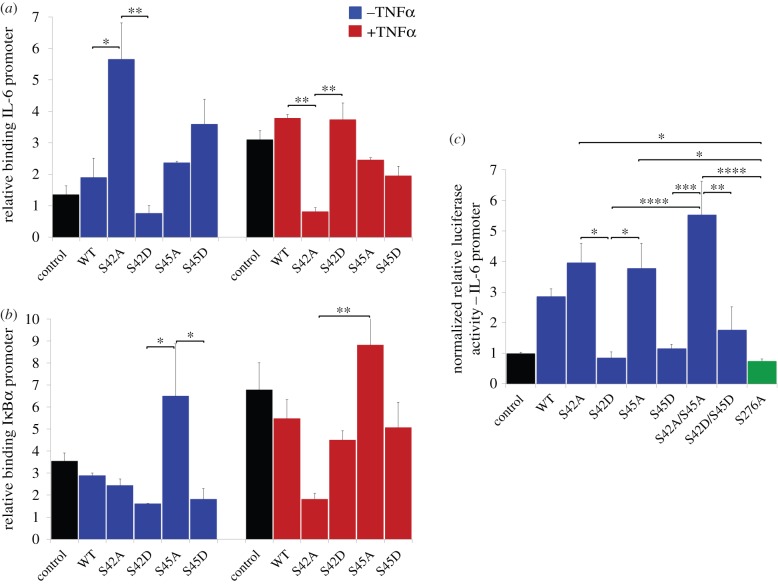
Ser42 and Ser45 regulate DNA binding and transcription. (*a*,*b*) SK-N-AS cells were either untransfected (control, black) or transfected with wild-type (WT) or mutant full-length RelA expression constructs. After 48 h, cells were treated with TNFα (5 ng ml^−1^ for 3 h) prior to cross-linking of chromatin and cell harvesting. Relative binding of RelA to the IL-6 (*a*) or IκBα (*b*) promoter was assessed by quantitative ChIP assay. (*c*) SK-N-AS cells were transfected with IL-6 promoter luciferase construct and co-transfected with WT or mutant full-length RelA as indicated. Cells were harvested after 48 h and luciferase assay carried out on cell lysates. Results are normalized to protein concentration; *n* = 3. A one-way analysis of variance (ANOVA) with a post hoc Tukey multiple comparisons test was used to probe for statistical differences: *p < 0.05, **p < 0.01, ***p < 0.001, ****p < 0.0001.

As a consequence of our molecular modelling, we hypothesized that phosphorylation of Ser42 and/or Ser45 regulates the interaction of RelA with κB DNA and RelA transcriptional activity. To examine potential roles for Ser42 and Ser45, both phosphomimetic (Ser → Asp) and phosphonull (Ser → Ala) mutants of RelA were assessed for their effect on RelA nuclear mobility (figure 5) and DNA binding (figure 6).

Fluorescent recovery after photobleaching (FRAP; figure 5*a*) was used to evaluate the nuclear mobility of RelA, under the hypothesis that differential DNA binding of the RelA protein variants would alter their diffusion rates. The recovery of fluorescent signal of RelA–DsRed after photobleaching is much slower for protein that is anchored to DNA (figure 5*b*,*c*); this assay is arguably of greater physiological relevance in evaluating the effect of modification of specific residues when compared with *in vitro* electromobility shift assay (EMSA). We observed no significant difference in the half time to recovery of any of the RelA–DsRed mutants from that of the wild-type (WT) protein in the cytoplasm of non-stimulated cells (figure 5*d*), as would be expected. However, there was a statistically significant difference in the nuclear dynamics of Ser42Asp and Ser45Asp mutants compared with WT in response to TNFα (figure 5*e*). Fluorescence signal of the RelA Ser42/45 phosphomimetics recovered much faster (lower *t*_half_) than that of WT protein after photobleaching, indicative of increased mobility of these two proteins under conditions where RelA is transcriptionally active and is thus expected to bind DNA. These data are in agreement with a significant decrease in DNA binding arising owing to phosphorylation at Ser42 or Ser45. As a control, recovery of fluorescent signal (a proxy for mobility) was also determined for a Tyr36Ala/Glu39Asp RelA non-DNA binding mutant (NBM) [47], which generated recovery curves similar to Ser42Asp and Ser45Asp (data not shown). Like the Ser42Ala/Ser45Ala proteins, the NBM only demonstrated a difference in mobility following TNFα stimulation. These data confirm an inhibitory effect of Ser42/45 phosphorylation on DNA binding, as predicted from our modelling. Interestingly, Ser42Ala–DsRed exhibited a small but statistically significant (*p* < 0.1) decrease in *t*_half_ after TNFα stimulation, suggesting a minor decrease in the ability of this mutant to remain anchored to chromatin. This may potentially be explained by the decreased capabilities of Ala compared with Ser to form hydrogen bonds with the DNA backbone [58].

To validate the roles of these RelA phosphorylation sites on cellular DNA binding in a more direct manner, chromatin immunoprecipitation (ChIP) studies were performed using either the IL-6 promoter (containing a single κB binding site—TGGGATTTTCCCA [59]) or the IκBα promoter (containing three distinct κB binding sites—GAGAAAGTCCCC, GGAAATTCCCC, GGGAAACCCC [60]) in SK-N-AS cells transfected with either WT RelA, Ser42 or Ser45 phosphonull or Ser42 or Ser45 phosphomimetic mutants (figure 6*a*). Owing to the presence of endogenous RelA in these ChIP assays, interpretation of these data has to consider co-precipitation of both endogenous and transfected RelA protein. In control cells, where endogenous RelA was expected to be largely unmodified at Ser42/45 (calculated as less than 20%, figure 4), there was a statistically significantly increase (2.9-fold) in IL-6 promoter binding of total RelA (endogenous and overexpressed) in cells transfected with Ser42Ala. No statistical difference was detected in RelA binding in cells expressing Ser45Ala RelA at this promoter region. As expected for conditions where RelA DNA binding was low, there was also no statistical difference in DNA binding in cells expressing the phosphomimetic mutants compared with those expressing WT RelA. In marked contrast, there was no difference in the binding of RelA from cells expressing Ser42Ala to the IκBα promoter, although increased binding of RelA to this promoter was observed in cells expressing the phosphonull Ser45Ala protein, suggesting a differential role for these two residues in mediating promoter-specific interactions.

Following cellular treatment with TNFα, where cells are undergoing active κB-mediated transcription, we confirmed enhanced DNA binding of RelA in the both the control and WT RelA transfected cells to both promoter sequences. Perhaps surprisingly, this stimulation-enhanced DNA binding of RelA was ablated at both promoter sequences upon transfection with the Ser42Ala RelA mutant. Indeed, there is a significant decrease in RelA binding following transfection of Ser42Ala upon stimulation, suggesting a dominant negative effect of Ser42Ala RelA under these conditions. Overexpression of Ser45Ala promotes a statistically significant increase in binding of RelA to the IκBα promoter, an effect not observed for the IL-6 promoter, once again indicating a differential effect of these two residues on promoter-specific binding.

To investigate the regulatory effects of phosphorylation at either of these two serine residues on transcription, SK-N-AS cells were transfected with an IL-6-promoter–luciferase reporter construct together with expression vectors for WT RelA, the single or double phosphonull or phosphomimetic mutants, or the known transcriptionally deficient Ser276Ala [39] RelA expression construct (figure 6*c*). Expression of the Ser42 and/or Ser45 phosphomimetics (S42D, S45D, S42D/45D) significantly repressed transcription from the IL-6 promoter, akin to that observed for Ser276Ala, when compared with the RelA phosphonull variants (S42A, S45A, S45A/S45A). However, no significant difference was observed between the two sites (Ser42 or Ser45) in terms of relative luciferase reporter activity, suggesting that phosphorylation at these sites may be partially redundant, at least in the context of the IL-6 promoter. These data therefore validate our hypothesis that phosphorylation of Ser42 and Ser45 abrogates DNA binding, consequently negatively impacting transcription.

A one-way analysis of variance (ANOVA) with a *post hoc* Tukey's multiple comparisons test was used to probe for statistical differences: *p < 0.05, **p < 0.01, ***p < 0.001 and ****p < 0.0001.

## 3. Discussion

In this study, we identified multiple novel phosphorylation sites on RelA, validating seven novel *in cellulo* phosphosites—Ser42, Ser131, Thr136, Ser238 (data not shown), Ser261, Ser269 and Ser472, and confirming Ser45 as a bona fide cellular site of regulated phosphorylation.

To the best of our knowledge, this is the most extensive, unbiased phosphosite analysis of an NF-κB transcription factor family member. For the first time, we have demonstrated distinct stimulation-induced temporal regulation of a number of phosphorylation events, and directly quantified the stoichiometry of TNFα-induced phosphorylation as a function of time for Ser42, Ser45 and Ser276 sites. Interestingly, the relative phosphorylation of many of these sites by purified protein kinases *in vitro* was dependent on the conditions for dimerization employed; modification of most sites was enhanced in the presence of the RelA heterodimerization partner p50, although PKA-mediated phosphorylation of Ser45 and Ser238, and IKKβ-mediated Thr136 phosphorylation were more efficient for RelA homodimers. Furthermore, with the exception of Ser42/45, phosphorylation was perturbed upon addition of IκBα, providing evidence for protein complex-specific effects on phosphorylation.

Putative functions for RelA phosphorylation sites were evaluated by bioinformatic and structural analysis, and new roles in regulating DNA binding predicted specifically for Ser42 and Ser45. In agreement with our hypothesis, Ser42 and Ser45 phosphomimetic versions of RelA exhibited increased nuclear mobility demonstrating decreased DNA anchoring, similar to that observed for the previously described RelA NBM (figure 5). Furthermore, the differential binding of RelA observed by ChIP upon transfection of the Ser42 and Ser45 mutants to either the IL-6 or IκBα promoters indicates a complex role for modification of these two residues in regulating promoter-specific interactions (figure 6). Based on a luciferase reporter assay, the inability to phosphorylate either or both of these residues promotes transcription from the IL-6 promoter, whereas RelA variants that mimic phosphorylation at Ser42 and/or Ser45 repress transcription from the same promoter. Interestingly, phosphorylation at Ser45 rather than at Ser42 appears to preferentially regulate binding to the IκBα promoter with a statistically significant increase in binding of the phosphonull Ser45Ala RelA. Curiously, Ser42Ala exhibits unexpected behaviour with respect to DNA binding at both promoters following cellular stimulation with TNFα. This may be owing to differential binding of other transcription factors to Ser42Ala, or potentially that Ser45 can still be phosphorylated in this Ser42Ala variant. These remain avenues for further exploration. It is interesting to note that *in vitro* phosphorylation of Ser45 by IKKβ (but not PKA) is significantly enhanced by p50, suggesting that this is heterodimer-specific phosphorylation event. The notable increase in phosphorylation at Ser42 and Ser45 upon inclusion of IκBα in the *in vitro* assay, and the close proximity of these two residues to the interface of RelA with IκB suggests a conformational change in this region that increases residue accessibility upon IκB binding. It will thus be interesting to evaluate the effect of modification of these sites on the conformation and interactome of RelA.

The dynamics of Ser42/45 phosphorylation is consistent with a model that modification of one or both of these residues serves to directly regulate RelA-mediated transcription by disrupting association with the κB promoter on DNA. For example, rapid phosphorylation 5 min after TNF exposure probably inhibits the function of RelA already localized to the nucleus, whereas the latter peak in pSer42/45 might act to prevent transcriptional activity after TNFα-induced RelA nuclear translocation. That pSer42 is never observed in the absence of pSer45 in response to this stimulus suggests that these residues may work together in a concerted ‘belt and braces’ mechanism to efficiently regulate DNA binding.

More generally, our *in vitro* kinase assays suggest a p50-induced change in the structure of RelA that facilitates its hyperphosphorylation. Abrogation of a number of these phosphorylation sites, particularly those localized to the C-terminal region of the RHD upon inclusion of IκBα, under conditions where native (inhibitor bound) RelA : p50 heterodimer is present, suggests either a masking effect by IκB on the otherwise available phosphorylation sites on RelA, or (more likely) a structural change in the heterodimer upon IκB binding which reduces accessibility of this region of the RHD. More generally, our data support the idea that different RelA dimers are probably subject to differential regulation and functional modulation.

Taken together, our biochemical, structural and cellular evaluation of RelA provides compelling evidence for a dynamic pool of post-translationally modified (primarily phosphorylated) proteoforms of RelA, which are rapidly and dynamically regulated in response to an extracellular stimulus (TNFα). The high complexity of the RelA phosphorylation patterning increases the likelihood of distinct combinations, or fingerprints, of protein modification (sometimes referred to as the NF-κB barcode hypothesis) [9,32] as one key for the formation of transcriptional complexes that support differential transcriptional output. While these studies focused primarily on phosphorylation of RelA at Ser42 and Ser45, it will be interesting to assess the dynamics of modification of these (and other) sites under different cellular conditions, with the ultimate view of identifying different RelA ‘codes’ or proteoforms that engage distinct promoters.

The inherent problems with ‘bottom-up’ peptide-based proteomics analyses are particularly acute for the comprehensive investigation of RelA (and other NF-κB) proteoforms. The analysis of the intact RelA protein by top-down proteomics [61,62] will therefore be particularly powerful when used in conjunction with our bottom-up discovery and targeted quantification strategies. The first top-down investigation using an overexpressed Halo-tagged form of RelA has recently been reported [63], although the myriad of phosphoforms that we know are present were not annotated, probably owing to the inherent difficulties of working on low abundance, highly modified protein species the size of RelA. Our new platform for RelA analysis should rapidly permit unique ‘NF-κB fingerprints’ and their functional relationships with promoter-specific transcription to be assessed.

## 4. Methods

### 4.1. Cell culture and lysis

SK-N-AS cells were grown in RPMI medium to 80% confluence and stimulated with TNFα (10 ng ml^−1^; Calbiochem) for up to 60 min. Growth medium was removed, and cells washed three times with PBS. Cells were lysed on ice by addition of lysis buffer (25 mM Tris–HCl pH 8, 0.15 M NaCl, 0.1% (v/v) NP-40, 1 mM EDTA, 1 mM EGTA, 10 mM β-glycerophosphate, 10 mM NaF, 300 µM Na_3_VO_4_, 1 mM benzamidine, 2 µM PMSF, mini EDTA-free protease inhibitor cocktail tablet and 1 mM DTT). Lysates were cleared by centrifugation (14 000*g*, 10 min, 4°C) and supernatants harvested. For the cross-linking IP experiments, the lysis buffer was modified to include 0.25% (v/v) Triton X-100 instead of 0.1% (v/v) NP-40 and 500 mM NaCl.

### 4.2. Immunoprecipitation of RelA using antibody cross-linked magnetic protein G beads

Cell lysates were pre-cleared by incubation with 20 µl of protein G beads (4°C for 10 min) followed by centrifugation (14 000*g*, 4°C, 1 min). RelA antibody (sc372, Santa Cruz Biotechnology; 2 µg) was cross-linked to magnetic protein G beads (20 µl) using the cross-linking magnetic IP kit (Pierce, USA) according to the manufacturer's instructions (see electronic supplementary material, Methods for full details). Cell lysate (500 µg) was added to the antibody-cross-linked beads and incubated for 1 h at room temperature. Unbound material was collected using a magnetic stand, beads were washed twice with 200 µl of lysis buffer, twice with 200 µl of IP buffer (40 mM Tris–HCl pH 8, 0.1% (v/v) NP-40, 1 mM EGTA, 6 mM EDTA, 6 mM DTT, 0.5 M NaCl, 1 mM benzamidine, 10 mM NaF, 10 mM β-glycerophosphate, 2 µM PMSF, 300 µM Na_3_VO_4_, mini EDTA-free protease inhibitor cocktail), once with 500 µl of HPLC water and three times with 500 µl of 25 mM NH_4_HCO_3_ pH 8. Beads were resuspended in 160 µl of 25 mM NH_4_HCO_3_, to which 10 µl of a 1% (w/v) solution of RapiGest SF [64] (Waters, UK) in 25 mM NH_4_HCO_3_ was added. Samples were heated at 96°C for 10 min, and supernatants containing the eluted RelA were recovered using a magnetic stand and used for in-solution digestion.

### 4.3. *In vitro* kinase assays

Purified truncated RelA (12–317; 5 µg [65]) was incubated with IKKα or PKA (Invitrogen, UK) in kinase buffer (25 mM Tris–HCl pH 7.5, 10 mM MgCl_2_, 0.5 mM EGTA, 2.5 mM DTT and 200 µM ATP) for 2 h at 37°C, 50 µl total volume. Assays were also performed with equimolar concentrations of p50 (35–381) [65], with or without equimolar GST-IκBα (59981, Abcam, UK). Phosphorylated proteins were diluted to 0.1 µg µl^−1^ in 50 mM NH_4_HCO_3_ and prepared for digestion.

### 4.4. Sample preparation for LC–MS/MS analysis

Disulfide bonds were reduced by addition of DTT (4.2 mM, 60°C, 10 min) and free Cys alkylated with iodoacetamide (IOA; 16 mM, dark, RT, 30 min). DTT was added to a final concentration of 8 mM and proteins digested by addition of trypsin (0.2 µg µl^−1^ in 10 mM AcOH, O/N, 37°C) to 2% (w/w). Digestion was stopped by addition of 5.5 µl 100% AcN and RapiGest SF hydrolysis induced with neat TFA (2.5 µl). Samples were incubated (37°C for up to 2 h, 400 r.p.m.) until a white precipitate was observed and then for a further 2 h at 4°C for 2 h. Insoluble hydrolysis product was removed by centrifugation (13 000*g*, 15 min, 4°C). Digestion was also performed without reduction and alkylation to preserve oxidative modifications of cysteine residues.

### 4.5. TiO_2_ enrichment of phosphorylated peptides

Digests were dried by centrifugal evaporation (10 µl) and resolubilized in loading buffer (65% (v/v) AcN, 2% (v/v) TFA, saturated with glutamic acid, 200 µl). Phosphopeptides were enriched using 200 µl pre-packed TiO_2_ spin tips (ProteaBio, France) [66]. Unbound peptides were dried by centrifugal evaporation, re-suspended in 20 µl of 0.1% (v/v) TFA and desalted using C_18_ Zip Tips (Millipore, USA). The eluates were dried as above, resolubilized in 200 µl of 2% (v/v) AcN, 0.1% (v/v) FA and dried again. After repeating the resuspension and drying, peptides were stored at −20°C for nano LC–ESI–MS/MS analysis.

### 4.6. Data-dependent nano LC–ESI–MS/MS

nLC–ESI–MS/MS analyses were performed on a Bruker amaZon ETD ion trap™ arranged in-line with an Ultimate 3000 nanoflow uHPLC system™ (Thermo Scientific, USA). Peptides were directly eluted over a 35 min LC gradient and a full scan mass spectrum acquired over *m/z* 300–1800, with the three most abundant ions being selected for isolation and activation with either CID and/or ETD (alternating). Peaklists from both the CID and ETD MS/MS spectra were extracted using Data Analysis software v. 4.0 (Bruker, Germany) and converted into mascot generic files (.mgf). .mgf files were searched against the concatenated SwissProt database (2011.05.03) and decoy using Mascot (v. 2.2.06), specifying *H. sapiens* for taxonomy. Parameters were set as follows: precursor mass tolerance at 0.4 Da; product ion tolerance at 0.6 Da; less than or equal to one missed cleavage; fixed modification: carbamidomethyl Cys; variable modifications: deamidation of Asn and Gln, oxidation of Met, phosphorylation of Ser, Thr and Tyr. Top-ranking peptide matches in Mascot were using ion score greater than or equal to 25 and an expectation value of less than or equal to 0.1. False-discovery rates at the protein and peptide level were assessed by searching against the decoy database (max. 1%). All CID and ETD tandem mass spectra of phosphorylated peptides were manually inspected.

### 4.7. Quantification of protein phosphorylation by selected reaction monitoring

Transitions for SRM were defined either using experimentally observed precursor and product ion *m/z* values of RelA (phospho)peptides, or Skyline (v. 1.2) [67] where this information was unavailable. All samples were analysed by nano uHPLC-SRM–MS/MS using a nanoACQUITY UPLC™ system in-line with a Xevo TQ MS (Waters, UK). Transition lists were divided to achieve a minimum dwell time of 50 ms per transition. Each peptide was defined by six transitions, including neutral loss of H_3_PO_4_ and (H_3_PO_4_ + H_2_O) for the phosphorylated peptides. Chromatographic traces of the transitions were manually inspected using Skyline (v. 1.2). A peptide was considered as positively identified when at least two co-eluting transitions could be observed. When available, retention times and relative abundance of product ions of *in vitro* generated RelA phosphopeptides were used as an additional filter. The most intense chromatographic peak of *n* co-eluting transitions for a single peptide (*n* = from 2 to 6) was classified as ‘top rank’ and its peak area value extracted.

### 4.8. Generation of pSer42/45 antibody

Phosphopeptide (and dephosphopeptide for purification) was synthesized corresponding to residues 38–50 (CEGRpSAGpSIPGER) where p indicates the sites of phosphorylation at Ser42 and Ser45. The phosphopeptide was used to generate a novel custom rabbit polyclonal antibody (Eurogentec).

### 4.9. Sequence alignment and interface analysis

RelA sequences were aligned using MUSCLE [26], and the conservation of phosphorylation sites was manually determined. Structural files containing RelA were recovered from the Protein Data Bank and protein : protein interfaces identified using Protein interfaces, surfaces and assemblies service (PISA; http://www.ebi.ac.uk/pdbe/prot_int/pistart.html [68]). Interfaces predicted to be the result of crystal artefacts were not analysed.

### 4.10. Fluorescence recovery after photobleaching

Cells were transfected using FuGENE HD (Promega) with RelA–dsRed-Express (dsRedxp) species and seeded in 2 ml of MEM (100 000 cells ml^−1^) in 35 mm single and four compartment glass base Greiner dishes and mounted in a Ziess XL incubator (37°C, 5% CO_2_). TNFα (10 ng ml^−1^) was added for 30 min to allow for a maximal first translocation prior to performing the FRAP analysis for the following 15 min. Images were collected using a Zeiss LSM 710 confocal microscope. dsRedXP was excited using 561 nm diode laser light, the resultant fluorescent emissions were collected between 580 and 630 nm, defining a 3.14 µm^2^ region of interest in the nucleus. Relative fluorescent intensity was normalized to the pre-bleached plateau with 100% corresponding to the highest recorded fluorescent intensity of a given cell and 0% corresponding to an absence of fluorescent signal. A ‘one-phase association, nonlinear regression’ curve ([*Y* = *Y*0 + (plateau – *Y*0) × (1 − exp(−*K* × *x*))], where *Y*0 is the *y*-value when *x* is zero, plateau is the *Y* value at infinite times and *K* is the rate constant) was fitted to the FRAP recovery curves; traces with an *R*^2^ value less than 0.7 were removed from the dataset. The derived rate constant (*K*) was used as a measure of nuclear mobility.

### 4.11. Cell transfection and chromatin immunoprecipitation assay

SK-N-AS cells were transfected in duplicate with 1 µg DsRed empty vector (control) or DsRed-RelA WT or mutant (including S42A, S42D, S45A, S45D) vectors using Effectene transfection reagent as per manufacturer's instructions (Qiagen). Cells were then grown for 48 h; one half of the duplicate cultures were treated with 5 ng ml^−1^ TNFα for 3 h and fixed in 1% formaldehyde, harvested by scraping into PBS with protease inhibitors and cell pellets resolubilized in lysis buffer (1% SDS, 10 mM EDTA, 50 mM Tris–HCl (pH 8.0)) for 30 min, then sonicated for 10 cycles (30 s on, 20 s off) in Diagenode Bioruptor. The sonicated samples were centrifuged (13 000 r.p.m., 4°C, 10 min), the cleared supernatant collected and chromatin content measured based on OD_260_ nm. Chromatin immunoprecipitation (ChIP) assays were carried out using 50 µg cross-linked pre-cleared chromatin which was incubated with 5 µg anti-RelA or control antibodies; the complexes were precipitated, washed and eluted. Cross-links were reversed and genomic DNA purified. Each PCR was performed in duplicate and the analysis repeated at least three times from independent ChIP experiments. A signal intensity value for each sample was calculated from the average of the experiments. Average values of eluates were normalized to average values of the control antibody sample and expressed as fold enrichment above background (i.e. control antibody). Quantitative PCR amplification was carried out using primers that encompass RelA binding site(s) within the human IL-6 or IκBα promoters (sequences available on request).

### 4.12. Luciferase reporter assay

SK-N-AS cells were transfected in triplicate with 100 ng of Renilla luciferase vector (pRLTK, transfection efficiency control, Promega), 0.5 µg IL-6 promoter–luciferase reporter (pIL6-Lux) and 1 µg RelA expression constructs, either WT or mutant (S42A, S42D, S45A, S45D, S42A/S45A, S42D/S45S, S276A, using Effectene transfection reagent as per the manufacturer's instructions (Qiagen). Luciferase assays were performed using a dual luciferase kit (Promega) according to the manufacturer's instructions. IL-6 promoter-driven expression of firefly luciferase was normalized for differences in transfection efficiency by measurement of the activity of the co-transfected Renilla luciferase vector (pRLTK). In brief, 48 h post-transfection cells were harvested into passive lysis buffer (part of dual luciferase kit). The firefly luciferase reagent (100 µl; LARII) was added to the test sample, with a 10 s equilibration time and measurement of luminescence with a 10 s integration time, followed by addition of 100 µl of the Renilla luciferase reagent and firefly quenching (Stop & Glo), 10 s equilibration time, and measurement of luminescence with a 10 s integration time. The data are represented as the ratio of firefly to Renilla luciferase activity (Fluc/Rluc).

## Supplementary Material

Supplementary Tables and Figures
