# Supplementary material for: Dynamic phosphorylation of RelA on Ser42 and Ser45 in response to TNFα stimulation regulates DNA binding and transcription
Source: Open Biol. 2016 Jul 27;6(7):160055. doi: 10.1098/rsob.160055 (PMC4967822; doi:10.1098/rsob.160055)
Supplement: Supplementary Tables and Figures [file rsob160055supp1.docx]

**Supplementary Table 1.** Phosphopeptides identified during data-dependent tandem MS analysis using either CID or ETD. Listed are the sequence, phosphorylation site identified, expected mass, mass difference, Mascot ion score, Mascot delta score, expectation value and charge state of all identified phosphorylated peptides.

| **Protein** | **Peptide** | **Site** | **Ref.** | **Discovery studies** | ***in vitro*** | **Expected mass (Da)** | **Mass difference (Da)** | **Ion score** | **Delta score** | **Expectation value** | **Charge state** | **CID/ETD** |
| --- | --- | --- | --- | --- | --- | --- | --- | --- | --- | --- | --- | --- |
| RelA/p65 | SAG**pS**IPGER | S45 | (1) | Y | Y | 952.14 | -0.26 | 53 | 23.8 | 2.40E-03 | 2 | CID |
| RelA/p65 | **pS**AG**pS**IPGER | S42/S45 | - | Y/Y |  | 1032.4 | -0.22 | 28 | 5.6 | 1.2E-1 | 2 | CID |
| RelA/p65 | CIH**pS**FQNLGIQCVK | S112 | - |  | Y | 1782.43 | -0.36 | 59 | 51.7 | 7.70E-04 | 3 | ETD |
| RelA/p65 | DLEQAI**pS**QR | S131 | - | Y | Y | 1138.22 | -0.31 | 48 | 19.8 | 8.30E-03 | 2 | CID |
| RelA/p65 | IQ**pT**NNNPFQVPIEEQR | T136 | - | Y | Y | 2005.54 | -0.38 | 78 | 62.7 | 1.60E-05 | 2 | ETD |
| RelA/p65 | N**pS**GSCLGGDEIFLLCDK | S203 | - |  | Y | 1963.44 | -0.36 | 106 | 12.3 | 2.10E-08 | 2 | CID |
| RelA/p65 | NSG**pS**CLGGDEIFLLCDK | S205 | (2, 3) |  | Y | 1963.44 | -0.36 | 101 | 0 | 7.90E-08 | 2 | ETD |
| RelA/p65 | G**pS**FSQADVHR | S238 | - |  | Y | 1182.16 | -0.32 | 55 | 25.9 | 1.10E-03 | 3 | ETD |
| RelA/p65 | GSF**pS**QADVHR | S240 | - |  | Y | 1182.16 | -0.32 | 39 | 1.1 | 5.80E-02 | 2 | CID |
| RelA/p65 | **pT**PPYADPSLQAPVR | T254 | (4) | Y |  | 1591.69 | -0.29 | 22 | 2.1 | 1.2E-01 | 2 | CID |
| RelA/p65 | TPPYADP**pS**LQAPVR | S261 | - | Y | Y | 1590.38 | -0.36 | 33 | 21.2 | 3.00E-01 | 2 | CID |
| RelA/p65 | TPPYADPSLQAPVRV**pS**MQLR | S269 | - | Y | Y | 2304.79 | -0.34 | 45 | 32.8 | 3.00E-02 | 3 | CID |
| RelA/p65 | EL**pS**EPMEFQYLPDTDDR | S281 | (2) |  | Y | 2163.48 | -0.39 | 29 | 13.7 | 7.3E-01 | 3 | CID |
| RelA/p65 | **pT**YETFK | T305 | - |  | Y | 867.12 | -0.22 | 18 | 9.3 | 1.2E-01 | 2 | CID |
| RelA/p65 | TYETFK**pS**IMK | S311 | (5) |  | Y | 1326.34 | -0.25 | 57 | 32.2 | 1.00E-03 | 3 | ETD |
| RelA/p65 | A**pS**VDD**pS**EFEQL | S468/S472 | (6, 7) | Y/Y |  | 1398.30 | -0.23 | 46 | 0 | 1.90E-03 | 2 | CID |
| p105 | LMFTAFLPD**pS**TGSFTR | S223 | - |  | Y | 1869.52 | -0.34 | 45 | 19.1 | 2.60E-02 | 2 | CID |
| p105 | LMFTAFLPDSTG**pS**FTR | S226 | - |  | Y | 1869.52 | -0.34 | 49 | 8.5 | 1.00E-02 | 2 | CID |
| p105 | RLEPVV**pS**DAIYDSK | S236 | - |  | Y | 1670.52 | -0.30 | 50 | 31.1 | 8.40E-03 | 2 | CID |
| p105 | TAGCV**pT**GGEEIYLLCDK | T263 | - |  | Y | 1964.48 | -0.34 | 74 | 25.2 | 3.00E-05 | 2 | CID |
| p105 | **pS**DLETSEPKPFLYYPEIK | S337 | (8) |  | Y | 2234.74 | -0.30 | 45 | 25.8 | 2.60E-03 | 3 | CID |
| IKKβ | HDSGLD**pS**MKDEEYEQMVK | S32 | - |  | Y | 2219.44 | -0.43 | 42 | 5.8 | 1.90E-03 | 3 | ETD |
| IKKβ | HD**pS**GLD**pS**MKDEEYEQMVK | S32/S36 | - |  | Y | 2299.39 | -0.45 | 44 | 26.2 | 2.10E-03 | 3 | CID |
| IKKβ | LEPQEVPRG**pS**EPWK | S63 | - |  |  | 1730.47 | -0.33 | 39 | 15.4 | 9.80E-02 | 3 | ETD |

**Supplementary Table 2.**  **Selected Reaction Monitoring (SRM) transitions of RelA peptides.** List of targeted peptides included in the SRM analysis. Sequences of peptides, including phosphorylation sites, are listed along with their respective precursor ions, precursor ion charge state, retention times (min) and product ions. C represents carbamidomethylated cysteine; -98 indicates loss of H_3_PO_4_ from the precursor ion; -116 indicates loss of (H_3_PO_4_+H_2_O) from precursor ion; -232 indicates loss of 2x(H_3_PO_4_+H_2_O) from precursor ion; n.d. - not detected; consistently observed product ions are highlighted in bold. Green reflects SRM assays for phosphopeptide quantification for which reproducible data was acquired; blue indicates the reference non-phosphorylated peptide.

| **Peptide** | **Site** | **Precursor ion m/z** | **Charge state** | **RT (min)** | **Product ions** |
| --- | --- | --- | --- | --- | --- |
| pSAGSIPGER | S42 | 477.20 | 2 | n.d. | -98; -116; y_8_; y_7_; y_7_Δ; y_5_ |
| SAGpSIPGER | S45 | 477.20 | 2 | 22.3 | **-98; 116**; y_7_; **y_7_Δ; y_5_**; y_4_ |
| pSAGpSIPGER | S42, S45 | 517.19 | 2 | 37.0 | **-**196;**-214**; y_8_; y_8_Δ; **y_5_; y_4_** |
| CIHpSFQNLGIQCVK | S112 | 592.27 | 2 | 34.5 | **-98**; **y_8_**; **y_7_**; **y_6_**; y_5_; **y_4_** |
| DLEQAIpSQR | S131 | 570.26 | 2 | 28.1 | **-98; -116**; y_7_; y_5_; **y_4_; y_3_** |
| IQpTNNNPFQVPIEEQR | T136 | 669.65 | 3 | 38.7 | -98; y_8_; **y_7_; y_6_; y_4_; y_10_^2+^** |
| VNRNSGpSCLGGDEIFLLCDK | S205 | 778.68 | 3 | 34.3 | **-98; y_18_^2+^; y_17_^2+^; y_16_^2+^; y_15_^2+^; y_14_^2+^** |
| GpSFSQADVHR | S238 | 592.25 | 2 | 21.1 | **-98; -116; y_9_Δ;** y_8_; **y_7_; y_6_** |
| GSFpSQADVHR | S240 | 592.25 | 2 | n.d. | -98; -116; y_8_; y_8_Δ; y_7_; y_7_Δ |
| pTPPYADPSLQAPVR | T254 | 796.38 | 2 | n.d. | -98; -116; y_13_; y_12_; y_11_; y_10_ |
| QVAIVFRpTPPYADPSLQAPVR | T254 | 802.42 | 3 | 34.1 | **-98; y_8_**; y_19_^2+^; y_18_^2+^; y_17_^2+^; y_16_^2+^ |
| TPPYADPpSLQAPVR | S261 | 796.38 | 2 | 35.2 | **-98**;- 116; y_12_Δ; y_9_; **y_8_**; y_8_Δ |
| QVAIVFRTPPYADPpSLQAPVR | S261 | 802.42 | 3 | n.d. | -98; y_11_; y_10_; y_7_; y_16_^2+^; y_15_^2+^ |
| pTPPYADPpSLQAPVR | T254, S261 | 836.36 | 2 | n.d. | -98; -196; y_10_; y_9_; y_8_; y_7_ |
| QVAIVFRpTPPYADpPSLQAPVR | T254, S261 | 829.07 | 3 | n.d. | -98; -196; y_8_; y_7_; y_15_^2+^; y_14_^2+^ |
| TPPYADPSLQAPVRVpSMQLR | S269 | 769.38 | 3 | 34.7 | **-98; -116**; y_10_; y_18_^2+^; **y_17_^2+^; y_14_^2+^** |
| VSMQLRRPpSDR | S276 | 475.56 | 3 | 26.3 | **-98**; y_7_; y_6_; **y_10_^2+^;** y_9_^2+^; y_8_^2+^ |
| RPpSDRELSEPMEFQYLPDTDDR | S276 | 926.07 | 3 | n.d. | -98; y_8_; y_4_; y_20_^2+^; y_19_^2+^; y_18_^2+^ |
| RPSDRELpSEPMEFQYLPDTDDR | S281 | 926.07 | 3 | n.d. | -98; y_8_; y_20_^2+^; y_19_^2+^; y_18_^2+^; y_15_^2+^ |
| ELpSEPMEFQYLPDTDDR | S281 | 722.29 | 3 | 33.4 | **-98**; -116; **y_13_^2+^;** y_10_^2+^; **y_12_^2+^;** y_8_; y_7_ |
| RPpSDRELpSEPMEFQYLPDTDDR | S276, S281 | 714.79 | 4 | n.d. | -98; -196; -232; y_11_; y_10_; y_9_ |
| pTIETFK | T305 | 434.68 | 2 | 32.0 | -**98**; -116; y_5_; **y_4_**; y_3_; **y_2_** |
| TYETFKpSIMK | S311 | 664.30 | 2 | 28.3 | **-98**; y_8_; -116; y_7_; y_6_; y_5_ |
| DGFYEAELCPDR | Reference | 736.31 | 2 | 36.2 | **y_9_; y_8_; y_7_; y_6_; y_5_; y_4_** |

**Supplementary Figure 1.** Product ion spectra generated either by CID or ETD of peptides from RelA/p65. The identified phosphorylation site is indicated and the sequence is detailed on the mass spectrum. (A) doubly charged ion at *m/z* 477.1 (CID): pSer45; (B) triply charged ion at *m/z* 595.1 (ETD): pSer112; (C) doubly charged ion at *m/z* 982.7 (CID): pSer203; (D) triply charged ion at *m/z* 655.5 (ETD): pSer205; (E) triply charged ion at *m/z* 395.1 (ETD): pSer238; (F) doubly charged ion at *m/z* 592.1 (CID): pSer240; (G) triply charged ion at *m/z* 557.9 (CID): pThr254 and pSer261; (H) doubly charged ion at *m/z* 722.3 (CID): pSer281; (I) doubly charged ion at *m/z* 434.6 (CID):pThr305; (J) triply charged ion at *m/z* 443.2 (ETD): pSer311; (K) doubly charged ion at *m/z* 467.1 (CID):pSer468 and pSer472 (CID).


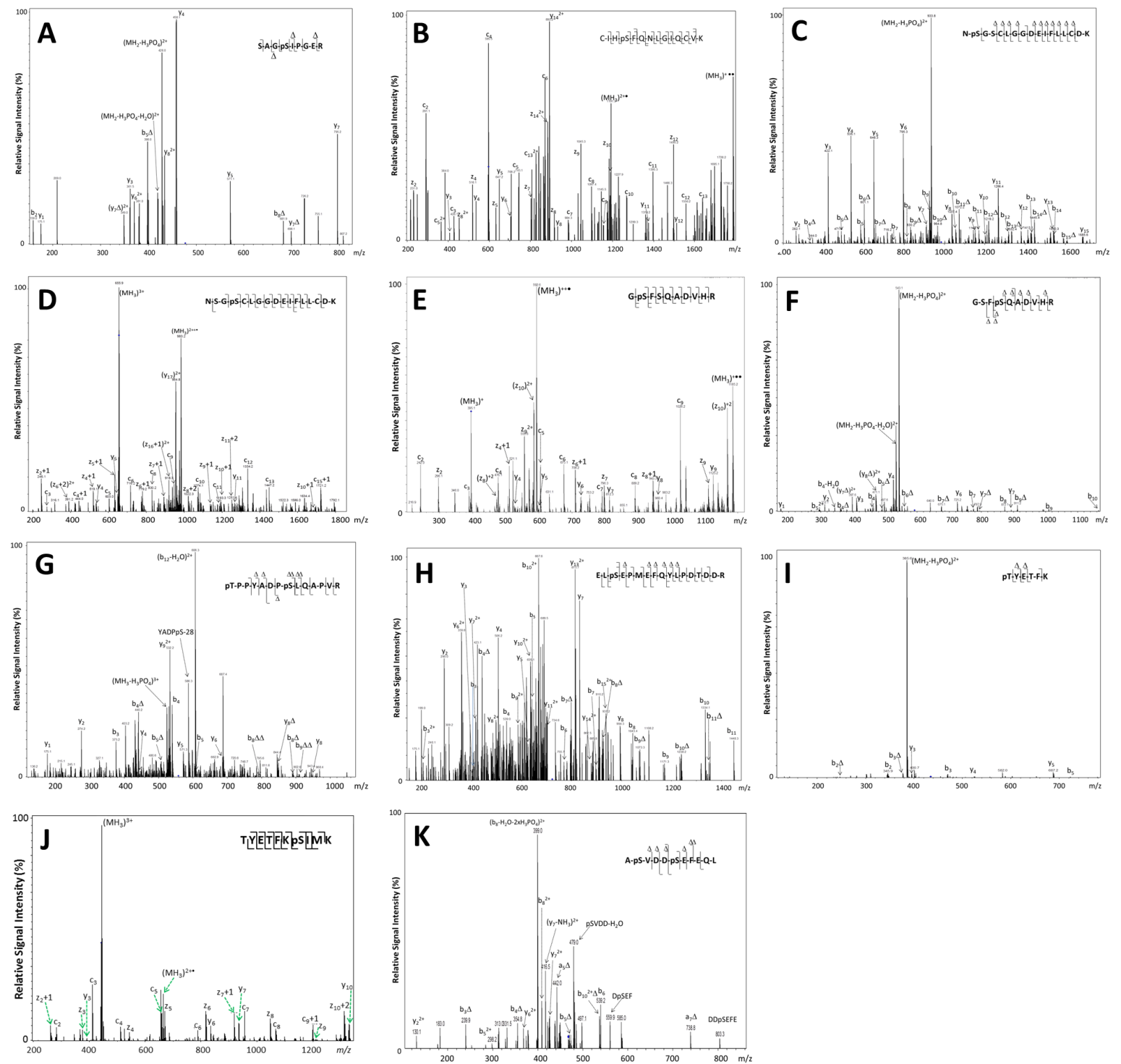


***In vitro* phosphorylation of IκBα**

As part of these studies, we were also able to assess the phosphorylation status of IκBα following the *in vitro* kinase assays. Incubation of the p65:p50:IκBα trimer with IKKβ promoted phosphorylation of IκBα on Ser32 and Ser36 (Supp. Fig. 2), supporting the role of IKKβ in mediating the phosphorylation event on IκBα that leads to ubiquitination and degradation of the inhibitory proteins, as occurs in the canonical activation pathway. PKA was also able to induce phosphorylation of IκBα on Ser32 in addition to phosphorylation of a novel site at Ser63 (Supp. Fig. 2).

**Supplementary Figure 2. Tandem mass spectra of IκBα tryptic phosphopeptides.** (A) Triply charged ion at *m/z* 740.8 indicating phosphorylation of Ser32 (ETD); (B) Triply charged ion at *m/z* 767.5 indicating phosphorylation of Ser32 and Ser36 (CID); (C) Triply charged ion at *m/z* 577.8 indicating phosphorylation of Ser63 (ETD).

***
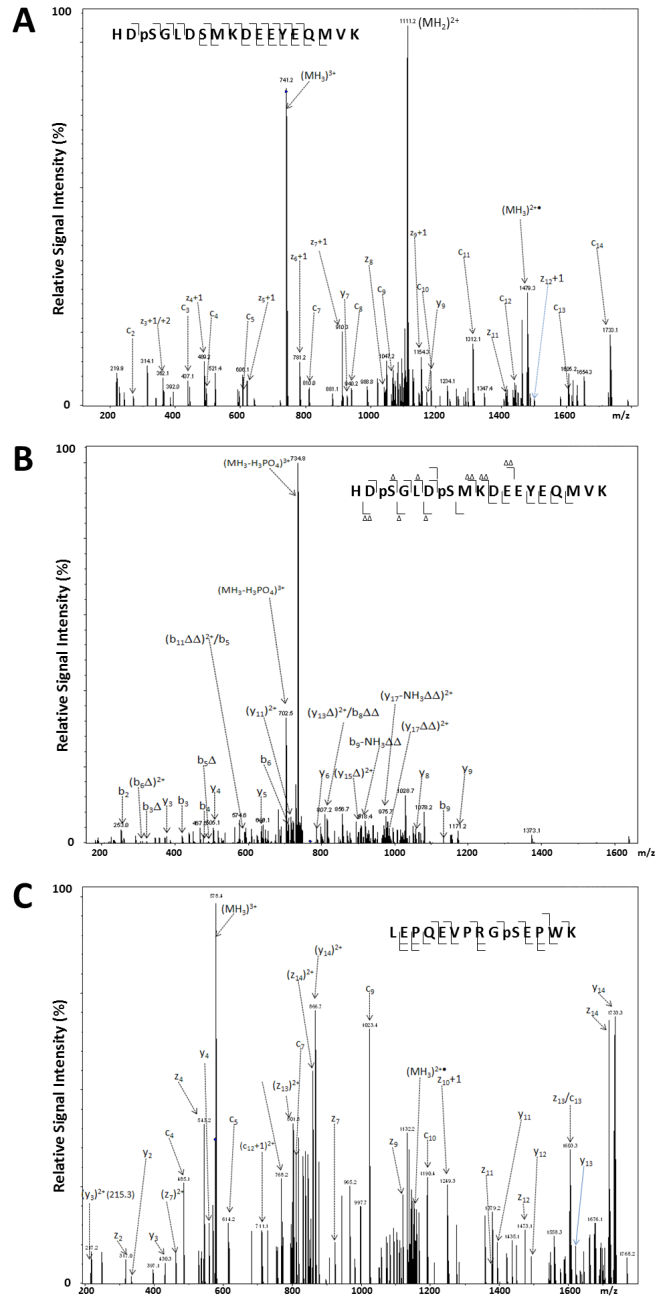
***

**Supplementary Figure 3. *In vitro* site-specific RelA phosphorylation is significantly increased in the presence of IκBα and/or p50.** The sites identified following *in vitro* phosphorylation of RelA with PKA or IKKβ, either alone or in the presence of stoichiometric amounts of p50, or p50 and IκBα are detailed. Selected reaction monitoring (SRM) determined relative change in phosphopeptide level compared to RelA alone with IKKβ. Statistical significance was assessed using a one-way ANOVA and a post-hoc Tukey test; * represents *p*<0.05; ** *p*<0.01; *** *p*<0.001 with respect to RelA alone for each site and kinase. # represents *p*<0.05; ## *p*<0.01; ### *p*<0.001 with respect to RelA:p50 for each site and kinase. N.B. Relative phosphorylation cannot be directly compared between phosphopeptides and thus phosphorylation sites due to differences in peptide ionisation efficiency.

**
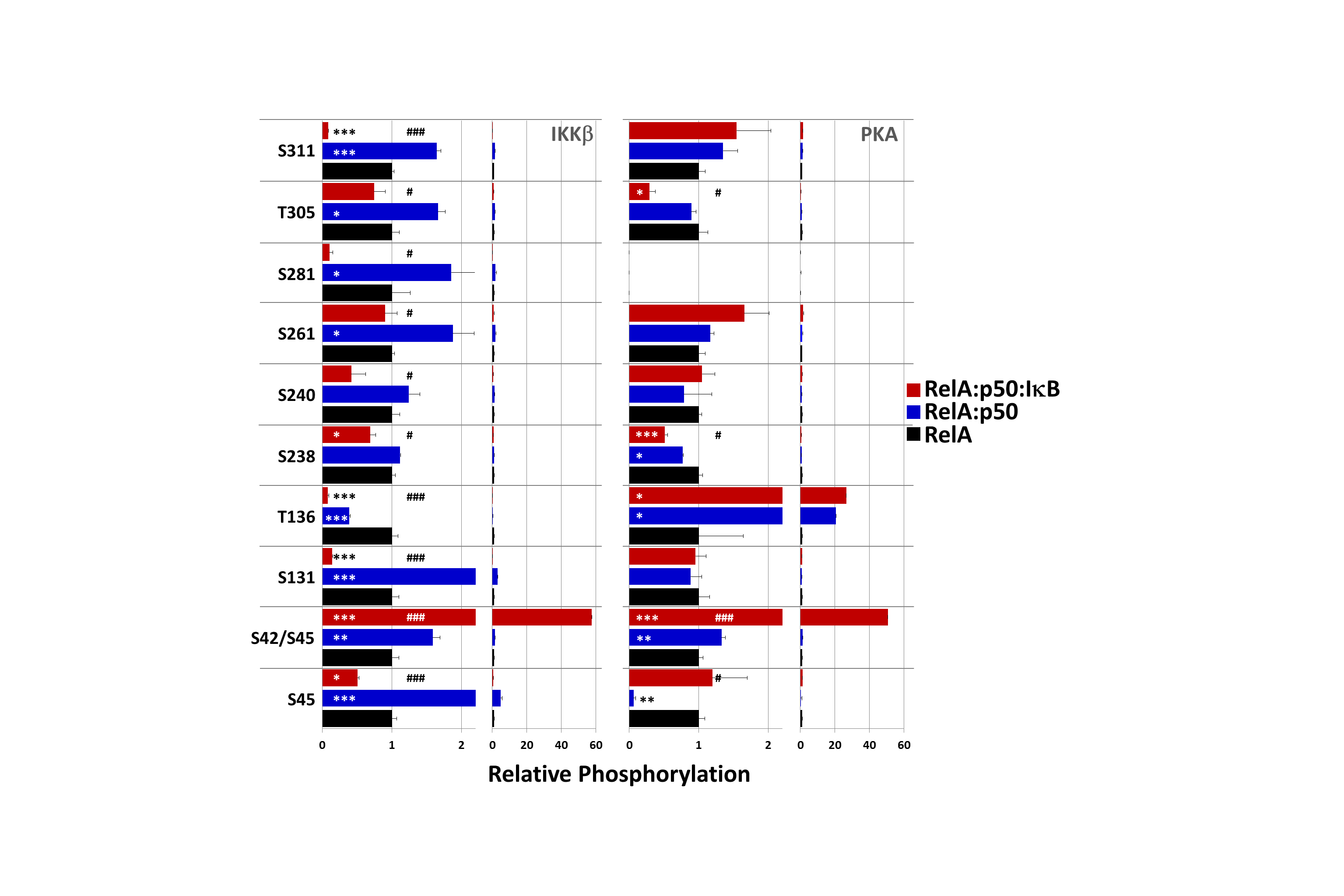
**

**Supplementary Figure 4. Cross-species sequence alignment of RelA/p65.** Thirteen higher eukaryote RelA/p65 protein sequences: Zebrafish (NP_001001839.2), Chicken (NP_990460.1), Xenopus laevis (NP_001081048.1), Opossum (XP_001379658.2), Mouse (NP_033071.1), Cow (NP_001073711.1), Pig (NP_001107753.1), Marmoset (XP_002807426.1), Rhesus macaque (AFE79532.1), Dog (XP_540850.2), Panda (XP_002916737.1), Horse (XP_001490867.3), Human (NP_068810.3), were aligned with MUSCLE. Sites of phosphorylation are highlighted red (serine) or yellow (threonine). Non-conservative substitutions are highlighted cyan.

NP_001001839.2|Zebrafish --MDGMFHQWGTSQ-----VPQGPPHVEIIEQPKSRGMRFRYKCEGRSAGSIPGEKSNDT

NP_990460.1|Chicken MEPADLLPLYLQPEWGEQEPGGATPFVEILEQPKQRGMRFRYKCEGRSAGSIPGEHSTDS

NP_001081048.1|Xenopus_laevis --MDGFHWTDIVSS-----MPPSIPPVEIIEQPKQRGMRFRYKCEGRSAGSIPGERSTDT

XP_001379658.2|Opossum --MEGLLPMIFASD----SPPARGPFVEIIEQPKQRGMRFRYKCEGRSAGSIPGERSTDT

NP_033071.1|Mouse --MDDLFPLIFPSE----PAQASGPYVEIIEQPKQRGMRFRYKCEGRSAGSIPGERSTDT

NP_001073711.1|Cow --MDDLFPLIFPAE----PAQASGPYVEIIEQPKQRGMRFRYKCEGRSAGSIPGERSTDT

NP_001107753.1|Pig --MDDLFPLIFPSE----PAPASGPYVEIIEQPKQRGMRFRYKCEGRSAGSIPGERSTDT

XP_002807426.1|Marmoset --MDELFPLIFPAE----PAQASGPYVEIIEQPKQRGMRFRYKCEGRSAGSIPGERSTDT

AFE79532.1|Rhesus_macaque --MDELFPLIFPAE----PAQASGPYVEIIEQPKQRGMRFRYKCEGRSAGSIPGERSTDT

XP_540850.2|Dog --MDDLFPLIFPAE----PAQASGPYVEIIEQPKQRGMRFRYKCEGRSAGSIPGERSTDT

XP_002916737.1|Panda --MADLFPLIFPSE----PAQASGPYVEIIEQPKQRGMRFRYKCEGRSAGSIPGERSTDT

XP_001490867.3|Horse --MEDLFPLIFPSE----PAQASGPYVEIIEQPKQRGMRFRYKCEGRSAGSIPGERSTDT

NP_068810.3|Human 1 --MDELFPLIFPAE----PAQASGPYVEIIEQPKQRGMRFRYKCEGRSAGSIPGERSTDT

: .. * ***:****.********************.*.*:

NP_001001839.2|Zebrafish TKTHPAIRVHNYSGPVRVRISLVTKNQPYKPHPHELVGKDCKHGYYEADL-QERRIHSFQ

NP_990460.1|Chicken ARTHPTIRVNHYRGPGRVRVSLVTKDPPHGPHPHELVGRHCQHGYYEAELSPERCVHSFQ

NP_001081048.1|Xenopus_laevis SKTHPTIKINNYQGPARIRISLVTKDSPHKPHPHELVGKDCKDGYYEAELSPDRSIHSFQ

XP_001379658.2|Opossum TKTHPTIKIHGYLGPGTVRISLVTKEPPHRPHPHELVGKDCRDGFYEAELCPDHSIHSFQ

NP_033071.1|Mouse TKTHPTIKINGYTGPGTVRISLVTKDPPHRPHPHELVGKDCRDGYYEADLCPDRSIHSFQ

NP_001073711.1|Cow TKTHPTIKINGYTGPGTVRISLVTKDPPHRPHPHELVGKDCRDGFYEAELCPDRCIHSFQ

NP_001107753.1|Pig TKTHPTIKINGYTGPGTVRISLVTKDPPHRPHPHELVGKDCRDGFYEAELCPDRCIHSFQ

XP_002807426.1|Marmoset TKTHPTIKINGYTGPGTVRISLVTKDPPHRPHPHELVGKDCRDGFYEAELCPDRCIHSFQ

AFE79532.1|Rhesus_macaque TKTHPTIKINGYTGPGTVRISLVTKDPPHRPHPHELVGKDCRDGFYEAELCPDRCIHSFQ

XP_540850.2|Dog TKTHPTIKINGYTGPGTVRISLVTKDPPHRPHPHELVGKDCRDGFYEAELCPDRCIHSFQ

XP_002916737.1|Panda TKTHPTIKINGYTGPGTVRISLVTKDPPHRPHPHELVGKDCRDGFYEAELCPDRCIHSFQ

XP_001490867.3|Horse TKTHPTIKINGYTGPGTVRISLVTKDPPHRPHPHELVGKDCRDGFYEAELCPDRCIHSFQ

NP_068810.3|Human 55 TKTHPTIKINGYTGPGTVRISLVTKDPPHRPHPHELVGKDCRDGFYEAELCPDRCIHSFQ

:.***:*.:: * ** :*:*****: *: ********. *. *:***:* :. :****

NP_001001839.2|Zebrafish NLGIQCVKKKDVGEAVSCRLQTQNNPFKIPDAKIWEEEFDLNAVRLCFQVSITL-SSGDL

NP_990460.1|Chicken NLGIQCVKKRELEAAVAERIRTNNNPFNVPMEER-GAEYDLSAVRLCFQVWVNG-PGG-L

NP_001081048.1|Xenopus_laevis NLGIQCVKKREVEDAVAHRIRTNNNPFNVSPEEL-KADYDLNTVCLCFQVFIPDQAAGRM

XP_001379658.2|Opossum NLGIQCVKKRDLEQAIAHRMQTNNNPFNVPLEEQ-RGDYDLNAVRLCFQVTIQD-SAGRP

NP_033071.1|Mouse NLGIQCVKKRDLEQAISQRIQTNNNPFHVPIEEQ-RGDYDLNAVRLCFQVTVRD-PAGRP

NP_001073711.1|Cow NLGIQCVKKRDLEQAISQRIQTNNNPFQVPIEEQ-RGDYDLNAVRLCFQVTVRD-PAGRP

NP_001107753.1|Pig NLGIQCVKKRDLEQAINQRIQTNNNPFQVPIEEQ-RGDYDLNAVRLCFQVTVRD-PAGRP

XP_002807426.1|Marmoset NLGIQCVKKRDLEQAINQRIQTNNNPFQVPIEEQ-RGDYDLNAVRLCFQVTVRD-PSGRP

AFE79532.1|Rhesus_macaque NLGIQCVKKRDLEQAITQRIQTNNNPFQVPIEEQ-RGDYDLNAVRLCFQVTVRD-PSGRP

XP_540850.2|Dog NLGIQCVKKRDLEQAISQRIQTNNNPFQVPIEEQ-RGDYDLNAVRLCFQVTVRD-PAGRP

XP_002916737.1|Panda NLGIQCVKKRDLEQAISQRIQTNNNPFQVPIEEQ-RGDYDLNAVRLCFQVTVRD-PAGRP

XP_001490867.3|Horse NLGIQCVKKRDLEQAISQRIQTNNNPFQVPIEEQ-RGDYDLNAVRLCFQVTVRD-PAGRP

NP_068810.3|Human 115 NLGIQCVKKRDLEQAISQRIQTNNNPFQVPIEEQ-RGDYDLNAVRLCFQVTVRD-PSGRP

*********.:: *: *:.*:****::. : ::**.:* ***** : ..*

NP_001001839.2|Zebrafish FPLEPVVSQPIYDNRAPNTAELKICRVNRNSGSCRGGDEIFLLCDKVQKEDIEVRFFLDS

NP_990460.1|Chicken CPLPPVLSQPIYDNRAPSTAELRILPGDRNSGSCQGGDEIFLLCDKVQKEDIEVRFWAEG

NP_001081048.1|Xenopus_laevis LPLPFVVSQPIYDNRAPNTAELKICRVNKNSGSCLGGDEIFLLCDKVQKEDIEVIFGLGN

XP_001379658.2|Opossum LVLPPVLSHPIYDNRAPNTAELKICRVNRNSGSCLGGDEIFLLCDKVQKEDIEVCFSGPG

NP_033071.1|Mouse LLLTPVLSHPIFDNRAPNTAELKICRVNRNSGSCLGGDEIFLLCDKVQKEDIEVYFTGPG

NP_001073711.1|Cow LRLAPVLSHPIFDNRAPNTAELKICRVNRNSGSCLGGDEIFLLCDKVQKEDIEVYFTGPG

NP_001107753.1|Pig LRLPPVLSHPIFDNRAPNTAELKICRVNRNSGSCLGGDEIFLLCDKVQKEDIEVYFTGPG

XP_002807426.1|Marmoset LRLLPVLSHPIFDNRAPNTAELKICRVNRNSGSCLGGDEIFLLCDKVQKEDIEVYFTGPG

AFE79532.1|Rhesus_macaque LRLPPVLSHPIFDNRAPNTAELKICRVNRNSGSCLGGDEIFLLCDKVQKEDIEVYFTGPG

XP_540850.2|Dog LRLSPVLSHPIFDNRAPNTAELKICRVNRNSGSCLGGDEIFLLCDKVQKEDIEVYFTGPG

XP_002916737.1|Panda LRLSPVLSHPIFDNRAPNTAELKICRVNRNSGSCLGGDEIFLLCDKVQKEDIEVYFTGPG

XP_001490867.3|Horse LRLSPVLSHPIFDNRAPNTAELKICRVNRNSGSCLGGDEIFLLCDKVQKEDIEVYFTGPG

NP_068810.3|Human 173 LRLPPVLSHPIFDNRAPNTAELKICRVNRNSGSCLGGDEIFLLCDKVQKEDIEVYFTGPG

* *:*:**:*****.****.* :.***** ******************* * .

NP_001001839.2|Zebrafish WESKGSFSQADVHRQVAIVFRTPPYCDTNLTEPLRVKMQLRRPSDREVSEPMDFQYLPSD

NP_990460.1|Chicken WEAKGSFAAADVHRQVAIVFRTPPFRERSLRHPVTVRMELQRPSDRQRSPPLDFRYLPHQ

NP_001081048.1|Xenopus_laevis WEARGIFSQADVHRQVAIVFRTPAFQDTKIRQSVKVQMQLRRPSDKEVSEPMEFQYLPDE

XP_001379658.2|Opossum WEARGSFSQADVHRQVAIVFRTPPYAEAALQAPVRVHMQLRRPSDRELSEPMEFQYLPDT

NP_033071.1|Mouse WEARGSFSQADVHRQVAIVFRTPPYADPSLQAPVRVSMQLRRPSDRELSEPMEFQYLPDT

NP_001073711.1|Cow WEARGSFSQADVHRQVAIVFRTPPYADPGLQAPVRVSMQLRRPSDRELSEPMEFQYLPDT

NP_001107753.1|Pig WEARGSFSQADVHRQVAIVFRTPPYADPSLQAPVRVSMQLRRPSDRELSEPMEFQYLPDT

XP_002807426.1|Marmoset WEARGSFSQADVHRQVAIVFRTPPYADPSLQAPVRVFMQLRRPSDRELSEPMEFQYLPDT

AFE79532.1|Rhesus_macaque WEARGSFSQADVHRQVAIVFRTPPYADPSLQAPVRVSMQLRRPSDRELSEPMEFQYLPDT

XP_540850.2|Dog WEARGSFSQADVHRQVAIVFRTPPYADPSLQAPVRVTMQLRRPSDRELSEPMEFQYLPDT

XP_002916737.1|Panda WEARGSFSQADVHRQVAIVFRTPPYADPSLQAPVRVSMQLRRPSDRELSEPMEFQYLPDT

XP_001490867.3|Horse WEARGSFSQADVHRQVAIVFRTPPYADPSLQAPVRVSMQLRRPSDRELSEPMEFQYLPDT

NP_068810.3|Human 233 WEARGSFSQADVHRQVAIVFRTPPYADPSLQAPVRVSMQLRRPSDRELSEPMEFQYLPDT

**:.* *: **************.: : : .: * *:*.****.: * *::*.***

NP_001001839.2|Zebrafish PDEHRLMEKRKRTEGMLHNLKLSSIITG----SSMSAE-RRPFPTAKRTLPVSKQPVAAS

NP_990460.1|Chicken GDLQCIEEKRKRTRDTFRAFVQRAPLPGLEPNPEPRPP-RRIAVPSR-----PP-PAPQQ

NP_001081048.1|Xenopus_laevis GDPHHIDEKRKRTLDNFKHYVKNNPFAG----GETRPQ-RRIAVANRNVPTKSE-PIRPS

XP_001379658.2|Opossum DDRHRIEEKRKRTLGTFKNIMKTSPFRG---NPDTWTSPRRIAVPSRTTGSTPKPPVPQP

NP_033071.1|Mouse DDRHRIEEKRKRTYETFKSIMKKSPFNG---PTEPRPPTRRIAVPTRNSTSVPK-PAPQP

NP_001073711.1|Cow DDRHRIEEKRKRTYETFKSIMKKSPFNG---PTDPRPPTRRIAVPNRGSASIPK-PAPQP

NP_001107753.1|Pig DDRHRIEEKRKRTYETFKSIMKKSPFNG---PTDPRPATRRIAVPSRSSASVPK-PAPQP

XP_002807426.1|Marmoset DDRHRIEEKRKRTYETFKSIMKKSPFNG---PTDPRPPPRRIAVPSRSSASIPK-PAPQP

AFE79532.1|Rhesus_macaque DDRHRIEEKRKRTYETFKSIMKKSPFSG---PTDPRPPPRRIAVPSRSSV-VPK-PAPQP

XP_540850.2|Dog DDRHRIEEKRKRTYETFKSIMKKSPFNG---PTDPRPPPRRIAVPSRSTTSVPK-PAPQS

XP_002916737.1|Panda DDRHRIEEKRKRTYETFKSIMKKSPFNG---PTDPRPPPRRIAVPSRSTPSVPK-PAPQP

XP_001490867.3|Horse DDRHRIEEKRKRTYETFKSIMKKSPFNG---PTDPRPPPRRIAVPARSSASVPK-PAPQP

NP_068810.3|Human 293 DDRHRIEEKRKRTYETFKSIMKKSPFSG---PTDPRPPPRRIAVPSRSSASVPK-PAPQP

* : : ****** :. : * . . ** . . . *

NP_001001839.2|Zebrafish AP--ASVPAV----------------SAAPPLKPPPTSFF-------------SPPPGQL

NP_990460.1|Chicken PP---------------------------SMVGAPPAPLFPLGVPPASSP---TPEP-LA

NP_001081048.1|Xenopus_laevis IP----------------------VPNPVVSCLPFSMPVLKAENVTSPST---LLSTVNI

XP_001379658.2|Opossum YTFPPPLSTINLEELSPIVFSASQV--QAPALASAPTPAP----SLVPAPTSTAPAPA--

NP_033071.1|Mouse YTFPASLSTINFDEFSPMLLPSGQISNQALALAPSSAPVL------AQTM---VPSSAMV

NP_001073711.1|Cow YSFTPSLSTINFEEFSPMVFPSGQIPSQTSALAPAPTPVLTQTQVLAPAP---APAPGMA

NP_001107753.1|Pig YPFTPSLSTINFDEFTPMAFASGQIPGQTSALAPAPAPVL----VQAPAP---APAPAMA

XP_002807426.1|Marmoset YPFSPSLNTINYDEFPTMVF----------------------------------------

AFE79532.1|Rhesus_macaque YPFTSSLSTINYDEFPTMVFPSGQI-SQASALA--PPQVL----PQAPAP---APAPAMV

XP_540850.2|Dog YPFTPSLSTINFEEFSPMVFSSGQISSQTSALASAPAPAP----ILAPAP----PAPA--

XP_002916737.1|Panda YPFTPSLSTINFEEFSPMVFPSGQIPNQTSALAPAPAPIL----AQAPAP---APAP---

XP_001490867.3|Horse YPFTPSLSTINFEEFSPMVFPSGTIPSQTSALAPAPAPVL------AQAP---APAPAMA

NP_068810.3|Human 349 YPFTSSLSTINYDEFPTMVFPSGQI-SQASALAPAPPQVL----PQAPAP---APAPAMV

.

NP_001001839.2|Zebrafish ----------------FTQQKMEPSPLPA-------------------------------

NP_990460.1|Chicken EALLQLQFDDGVGGSGPPPSTTTTTTTTQCALGGGIPDP-----GGSPLDLGALLGDPP-

NP_001081048.1|Xenopus_laevis SDFSN-----------LGFSSQPPSQSDHDRLESMLNYP--SFPGDANLDLVEMLPHENE

XP_001379658.2|Opossum ----------------------PALPPISQSGEGTLSEALLGLQFDTDGDLAEILADPD-

NP_033071.1|Mouse -PLAQPPAPAPVLTPGPPQSLSAPVPKSTQAGEGTLSEALLHLQFDADEDLGALLGNSTD

NP_001073711.1|Cow STLAQA------LAPGLAQAVTPPAPRTNQTGEGTLTEALLQLQFDTDEDLGALLGNNTD

NP_001107753.1|Pig SALAQAPAPVPVLAPGLAQAVAPPAPKTNQAGEGTLTEALLQLQFDTDEDLGALLGNNTD

XP_002807426.1|Marmoset -----------------------------------------------------LLGNSTD

AFE79532.1|Rhesus_macaque SPLAQA----PVLAPGPPQAVAPPAPKPTQAGEGTLSEALLQLQFD-DEDLGALLGNSTD

XP_540850.2|Dog --------PAPILAPGLAQAMAPPAPKTTQAGEGTLTEALLQLQFDADEDLGALLGNSAD

XP_002916737.1|Panda ----------------------PPIPKTTQAGEGTLSEALLHLQFDADEDLGALLGNSTD

XP_001490867.3|Horse SALAQAPAPVPVLAAGLAQAVAPPAPRTTQAGEGTLTEALLQLQFDADEDLGALLGNNTD

NP_068810.3|Human 401 SALAQAPAPVPVLAPGPPQAVAPPAPKPTQAGEGTLSEALLQLQFD-DEDLGALLGNSTD

NP_001001839.2|Zebrafish -------SSSDIWKYLQAMS--------------------------VDSQ----------

NP_990460.1|Chicken ------FDTIDAAELQRLLGPPETPPGGIGAGGGFGELLSLPTNFGDPPSSTAATFGPSP

NP_001081048.1|Xenopus_laevis SRC-TSLSSIDNSDFSQLLSESQS--------------SGTLSAALQEPG-------TSQ

XP_001379658.2|Opossum -STYTNLAAIDNSEFQQLLN------------------QGIPGTLEGPGP-------SGE

NP_033071.1|Mouse PGVFTDLASVDNSEFQQLLN------------------QGVS----MSHS-------TAE

NP_001073711.1|Cow PAVFTDLASVDNSEFQQLLN------------------QGVP----MGPH-------TAE

NP_001107753.1|Pig PTVFTDLASVDNSEFQQLLN------------------QGVS----MPPH-------TAE

XP_002807426.1|Marmoset PAVFTDLASVDNSEFQQLMN------------------QSLP----VAPH-------TAE

AFE79532.1|Rhesus_macaque PTVFTDLASVDNSEFQQLLN------------------QGVP----VAPH-------TTE

XP_540850.2|Dog PAVFTDLASVDNSEFQQLLN------------------QGVS----VAPH-------TAE

XP_002916737.1|Panda PAVFTDLASVDNSEFQQLLN------------------QGVS----VAPH-------TAE

XP_001490867.3|Horse PAVFTDLASVDNSEFQQLLN------------------QGVS----MAPH-------TAE

NP_068810.3|Human 460 PAVFTDLASVDNSEFQQLLN------------------QGIP----VAPH-------TTE

: * . . :.

NP_001001839.2|Zebrafish ------PKAV----------------PVLPFPSGTVST----GRDARLITAARGENTVLH

NP_990460.1|Chicken PMLLSYPEAITRLVQCQTPGGSGGGGPPVGPPQDLGGPLHPPGAPPQP-----TEDSLPS

NP_001081048.1|Xenopus_laevis GTFMAYPESIARLM-------T--NRPNEDEGGERIDS----GLINGMFDISREEIHLTS

XP_001379658.2|Opossum PMLMEYPESITRLM-------TGSQRPPEPTPAPPGAS----GLANGLLG---ADEVFPS

NP_033071.1|Mouse PMLMEYPEAITRLV-------TGSQRPPDPAPTPLGTS----GLPNG-LS---GDEDFSS

NP_001073711.1|Cow PMLMEYPEAITRLV-------TGSQRPPDPAPTPLGPP----GLTNGLLS---GDEDFSS

NP_001107753.1|Pig PMLMEYPEAITRLV-------TGSQRPPDPAPTPLGAS----GLTNGLLS---GDEDFSS

XP_002807426.1|Marmoset PMLMEYPEAITRLV-------TGAQRPPDPAPAPLGAP----GLPNGLLS---GDEDFSS

AFE79532.1|Rhesus_macaque PMLMEYPEAITRLV-------TGAQRPPDPAPAPLGAP----GLPNGLLS---GDEDFSS

XP_540850.2|Dog PMLMEYPEAITRLV-------TGSQRPPDPVPAPVGAS----GLPNGLLS---GDEDFSS

XP_002916737.1|Panda PMLMEYPEAITRLV-------TGSQRPPDPAPAPLGAS----GLPNGLLS---GDEDFSS

XP_001490867.3|Horse PMLMEYPEAITRLM-------SGSQRPPDPAPAPLGPX----GLPNGLLS---GDEDFSS

NP_068810.3|Human 491 PMLMEYPEAITRLV-------TGAQRPPDPAPAPLGAP----GLPNGLLS---GDEDFSS

*::: * * : .

NP_001001839.2|Zebrafish PYTLHYTHLTHVLLLV

NP_990460.1|Chicken LGDLDFSAFLSQFPSS

NP_001081048.1|Xenopus_laevis LFELDFSSLLSNMK--

XP_001379658.2|Opossum MGDLDISAFLSQISS-

NP_033071.1|Mouse IADMDFSALLSQISS-

NP_001073711.1|Cow IADVDFSALLSQISS-

NP_001107753.1|Pig IADMDFSALLSQISS-

XP_002807426.1|Marmoset IADMDFSALLSQISS-

AFE79532.1|Rhesus_macaque IADMDFSALLSQISS-

XP_540850.2|Dog IADMDFSALLSQISS-

XP_002916737.1|Panda IADMDFSALLSQISS-

XP_001490867.3|Horse ISDMDFSALLSQISS-

NP_068810.3|Human 537 IADMDFSALLSQISS-

: : : :

**Supplementary Figure 4.** Example SRM chromatograms of the control peptide DGFTEAELCPDR (A) and the phosphopeptide SAGpSIPGER [pS45] (B). Prec-98 refers to neutral loss of H_3_PO_4_ from the precursor ion; Prec-116 refers to neutral loss of (H_3_PO_4_+H_2_O) from the precursor ion.

**A**


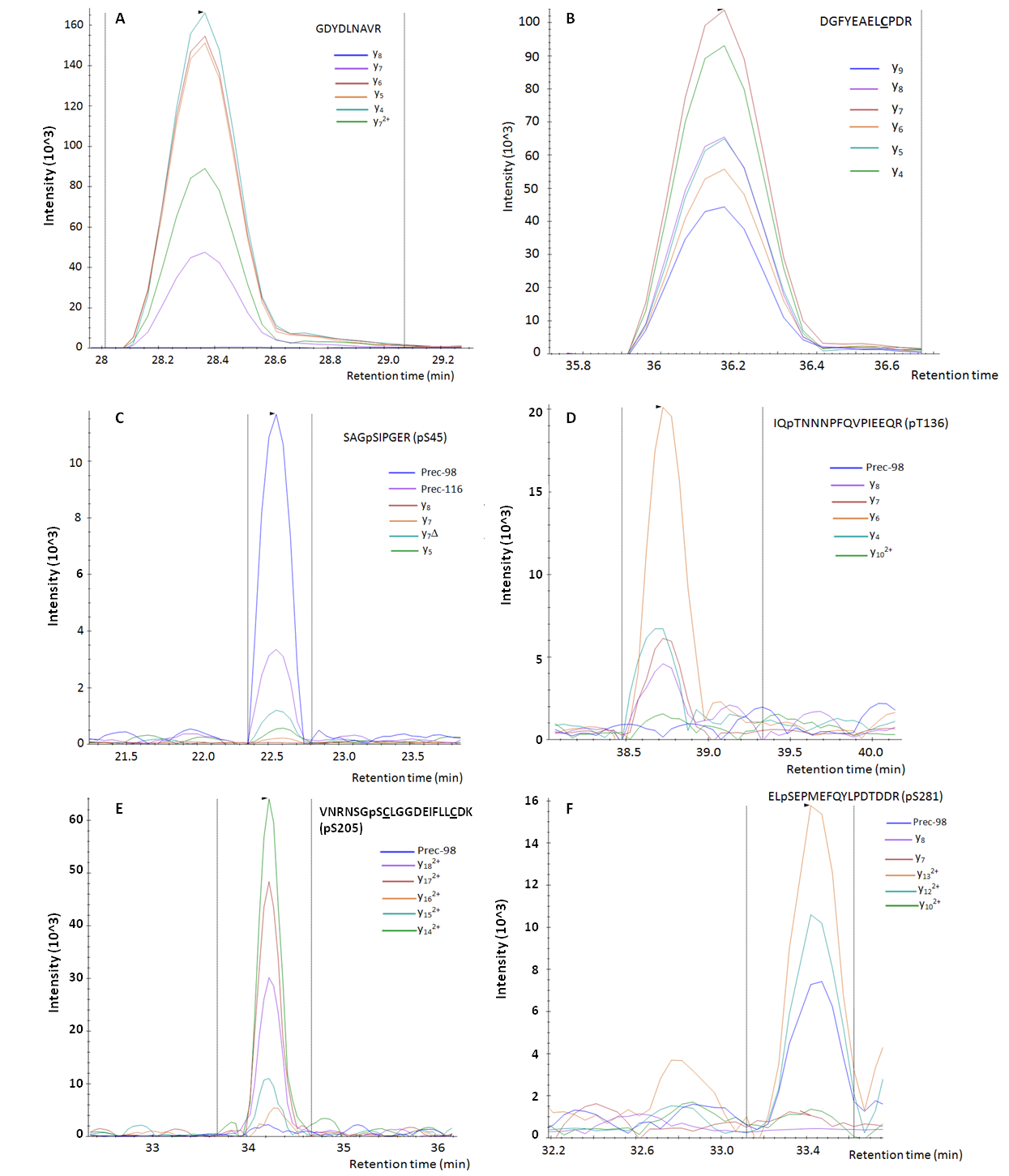

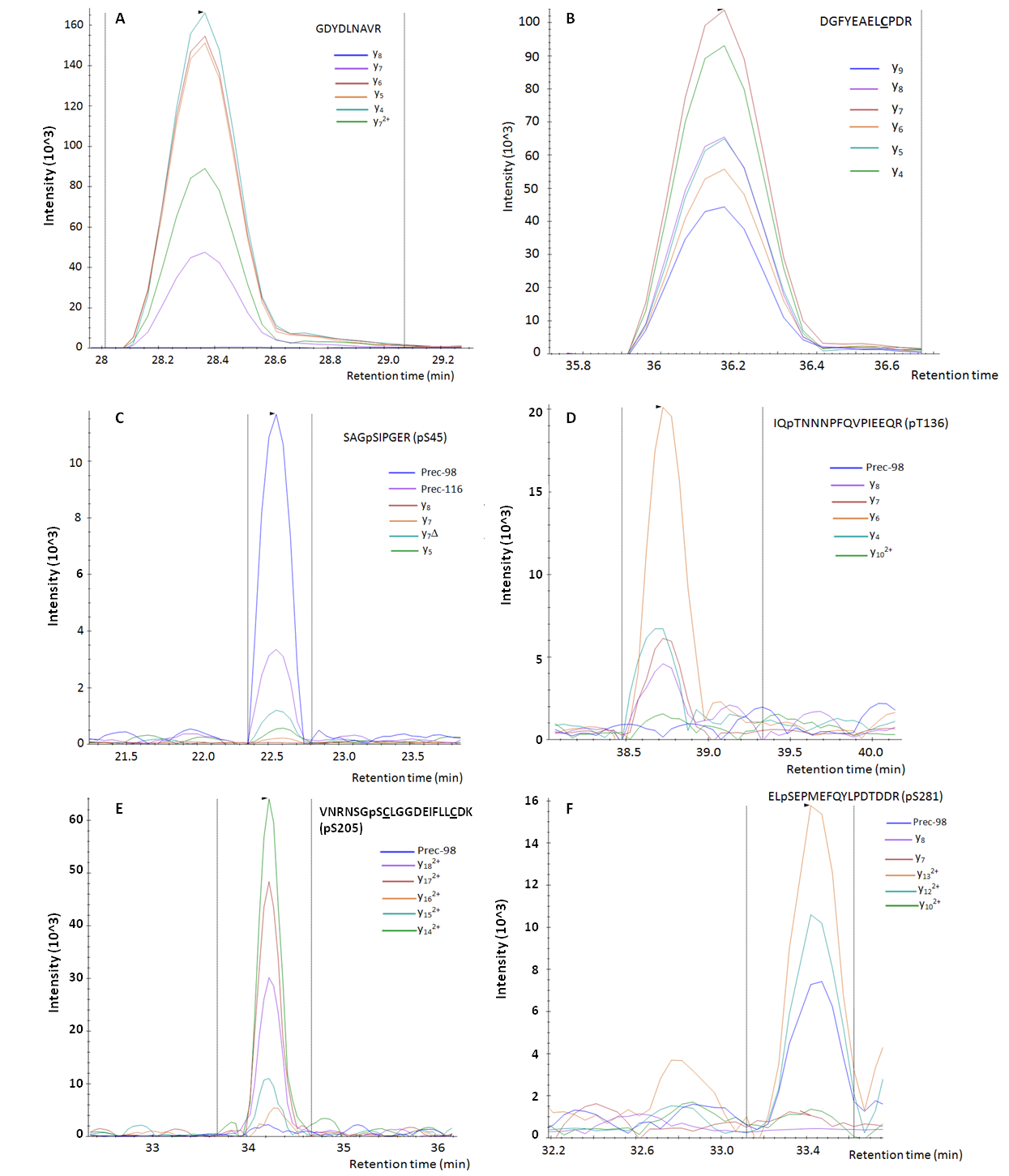


**B**

**Supplementary Figure 5.** Quality control of the SRM transitions for the RelA/p65 reference peptide DGFYEAELCPDR as a function of time post-TNFα stimulation: Depicted are the relative peak areas (top) and the measured retention time (min) (bottom) for each of the transitions. RSD of retention times are 0.14%.

**
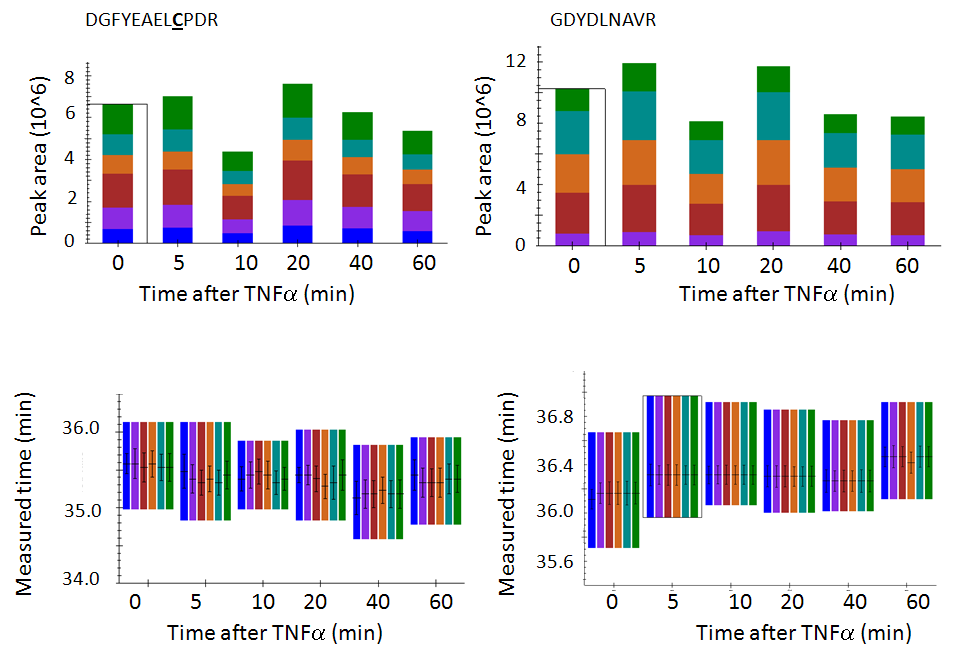
**

1. Sharma K, D'Souza RC, Tyanova S, Schaab C, Wisniewski JR, Cox J, et al. Ultradeep human phosphoproteome reveals a distinct regulatory nature of Tyr and Ser/Thr-based signaling. Cell Rep. 2014;8(5):1583-94.

2. Anrather J, Racchumi G, Iadecola C. cis-acting, element-specific transcriptional activity of differentially phosphorylated nuclear factor-kappa B. J Biol Chem. 2005;280(1):244-52.

3. Hochrainer K, Racchumi G, Anrather J. Hypo-phosphorylation leads to nuclear retention of NF-kappaB p65 due to impaired IkappaBalpha gene synthesis. FEBS Lett. 2007;581(28):5493-9.

4. Ryo A, Suizu F, Yoshida Y, Perrem K, Liou YC, Wulf G, et al. Regulation of NF-kappaB signaling by Pin1-dependent prolyl isomerization and ubiquitin-mediated proteolysis of p65/RelA. Mol Cell. 2003;12(6):1413-26.

5. Duran A, Diaz-Meco MT, Moscat J. Essential role of RelA Ser311 phosphorylation by zetaPKC in NF-kappaB transcriptional activation. EMBO J. 2003;22(15):3910-8.

6. Mattioli I, Sebald A, Bucher C, Charles RP, Nakano H, Doi T, et al. Transient and selective NF-kappa B p65 serine 536 phosphorylation induced by T cell costimulation is mediated by I kappa B kinase beta and controls the kinetics of p65 nuclear import. J Immunol. 2004;172(10):6336-44.

7. Buss H, Dorrie A, Schmitz ML, Frank R, Livingstone M, Resch K, et al. Phosphorylation of serine 468 by GSK-3beta negatively regulates basal p65 NF-kappaB activity. J Biol Chem. 2004;279(48):49571-4.

8. Hou S, Guan H, Ricciardi RP. Phosphorylation of serine 337 of NF-kappaB p50 is critical for DNA binding. J Biol Chem. 2003;278(46):45994-8.
